# Synthetic Scientific Image Generation with VAE, GAN, and Diffusion Model Architectures

**DOI:** 10.3390/jimaging11080252

**Published:** 2025-07-26

**Authors:** Zineb Sordo, Eric Chagnon, Zixi Hu, Jeffrey J. Donatelli, Peter Andeer, Peter S. Nico, Trent Northen, Daniela Ushizima

**Affiliations:** 1 Applied Math and Computational Research Division, Lawrence Berkeley National Laboratory, Berkeley, CA 94720, USA; zsordo@lbl.gov (Z.S.); echagnon@lbl.gov (E.C.); zixihu@lbl.gov (Z.H.); jjdonatelli@lbl.gov (J.J.D.); 2 Environmental Genomics and Systems Biology Division, Lawrence Berkeley National Laboratory, Berkeley, CA 94720, USA; pfandeer@lbl.gov (P.A.); trnorthen@lbl.gov (T.N.); 3 Earth Sciences Division, Lawrence Berkeley National Laboratory, Berkeley, CA 94720, USA; psnico@lbl.gov; 4 Bakar Computational Health Sciences Institute, University of California, San Francisco, CA 94158, USA; 5 Berkeley Institute for Data Science, University of California, Berkeley, CA 94720, USA

**Keywords:** image generation, generative AI, Generative Adversarial Networks, diffusion, synthetic data

## Abstract

Generative AI (genAI) has emerged as a powerful tool for synthesizing diverse and complex image data, offering new possibilities for scientific imaging applications. This review presents a comprehensive comparative analysis of leading generative architectures, ranging from Variational Autoencoders (VAEs) to Generative Adversarial Networks (GANs) on through to Diffusion Models, in the context of scientific image synthesis. We examine each model’s foundational principles, recent architectural advancements, and practical trade-offs. Our evaluation, conducted on domain-specific datasets including microCT scans of rocks and composite fibers, as well as high-resolution images of plant roots, integrates both quantitative metrics (SSIM, LPIPS, FID, CLIPScore) and expert-driven qualitative assessments. Results show that GANs, particularly StyleGAN, produce images with high perceptual quality and structural coherence. Diffusion-based models for inpainting and image variation, such as DALL-E 2, delivered high realism and semantic alignment but generally struggled in balancing visual fidelity with scientific accuracy. Importantly, our findings reveal limitations of standard quantitative metrics in capturing scientific relevance, underscoring the need for domain-expert validation. We conclude by discussing key challenges such as model interpretability, computational cost, and verification protocols, and discuss future directions where generative AI can drive innovation in data augmentation, simulation, and hypothesis generation in scientific research.

## 1. Introduction

Generative AI (genAI) has rapidly advanced as a powerful tool to synthesize new digital content, including images, text, and music [1,2]. Although these models have achieved remarkable success in generating high-quality visuals for artistic and commercial use, their application to scientific imaging presents significant challenges. In particular, generating accurate images of scientific phenomena that were not represented in the training data often results in hallucinations [3] or misrepresentations of fundamental physical and biological principles [4]. Such failures can result in visually convincing but scientifically implausible outputs, potentially propagating misconceptions, contributing poor images to training sets, and hindering scientific progress [5,6].

This article investigates generative image modeling within two primary subdomains: text-to-image and image-to-image generation. We begin with a comprehensive overview of recent key developments, followed by an in-depth discussion of how leading architectures such as Variational Autoencoders (VAEs), Generative Adversarial Networks (GANs), and Diffusion Models have revolutionized areas reliant on image analysis. Particular emphasis is placed on their applications in scientific contexts, where precision, fidelity, and interpretability are paramount. Thus, we discuss strategies and methodologies for the verification and validation of synthetic images.

The main contributions of this paper are:

- Detailed analysis of generative methods for text-to-image and image-to-image synthesis, with emphasis on scientific relevance.
- Comparative evaluation of generative architectures, highlighting their capabilities and limitations across multiple scientific domains and material types.
- Critical discussion of current challenges and future directions, including pathways for verifying and validating the scientific integrity of synthetic images.

## 2. Background

Image-generating models have become a prevalent area of research in recent years, fueled by advances in both algorithm design and hardware capabilities. Early efforts focused on conventional data-augmentation techniques such as rigid-body transformations [7], but the field has since evolved toward more sophisticated approaches, including the development of foundational models [8].

**Figure 1:**
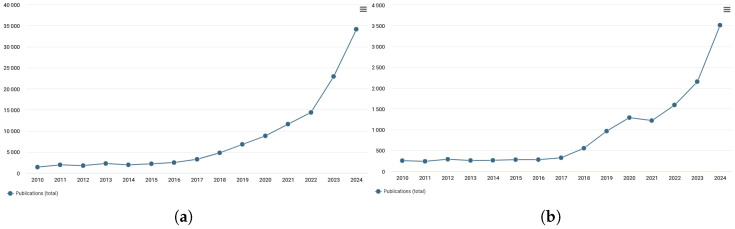
Publications of image-generation papers over the last 15 years. (**a**) Publication trends collected from all public sources. (**b**) Publication trends excluding those from Arxiv.

To illustrate the accelerating interest in image generation, Figure 1 presents publication trends over the past decade, using data from Dimensions [9]. After remaining steady in the early 2010s, the number of publications began to rise sharply around 2017. This growth reflects a convergence of factors: breakthroughs in generative algorithms, expanded access to large-scale datasets, and the proliferation of high-performance computing resources. Given this pace of innovation, it is key to critically assess the applicability and limitations of these models, particularly within scientific imaging contexts.

During the earlier phase of this acceleration, Generative Adversarial Networks (GANs) dominated the field, especially in image synthesis tasks. The significant increase in the number of publications can be attributed to specific breakthroughs that both enabled and inspired future work in Image Generation. Key breakthroughs in 2017 and 2018 significantly advanced GAN performance, yielding state-of-the-art (SOTA) results. For example, Pix2Pix [10] introduced conditional adversarial networks for image-to-image translation, enabling the model to learn mappings from input to output images using paired datasets. CycleGAN [11] further extended this approach by allowing image translation from unpaired data, a key feature for real-world applications where aligned datasets are scarce or unavailable. These innovations laid the foundation for powerful image manipulation tools, such as style transfer and background replacement.

Introduced in 2018, StyleGAN [12] redefined the field by introducing an alternative generator architecture that enabled unprecedented control over the latent space, allowing for fine-grained manipulation of image attributes. This shift brought significant improvements in visual quality and consistency. A year later, transformers [13] entered the generative space with SAGAN (Self-attention GAN) [14], which introduced self-attention layers into both the generator and discriminator, allowing the models to account for long-range dependencies in the image. Building on this, BigGAN scaled up GANs substantially, resulting in higher-resolution outputs with greater diversity, albeit with a corresponding increase in computational demands.

The year 2020 marked the emergence of Diffusion Models as competitive alternatives to GANs. Initially introduced by Sohl-Dickstein et al. [15], these models iteratively add and remove noise from images in a learned forward-reverse process, achieving state-of-the-art results on high-resolution tasks and rivaling the performance of top GANs. In parallel, transformer-based architectures, originally designed for language modeling [13], began to influence vision research, particularly with Vision Transformer (ViT), which reframed image classification by treating image patches as input tokens, similarly to natural language processing [13]. Building upon this work, Ho et al. create their Denoising Diffusion Probabilistic Models (DDPM) [16], which formulate new strategies for training and sampling from Diffusion Models. This resulted in SOTA performance compared to other Diffusion Models, and similar performance to SOTA GANs on higher-resolution images.

Transformers also fueled advances in text-to-image generation. In 2021, OpenAI introduced DALL-E [17], a transformer-based model that synthesized coherent and often whimsical images from textual prompts. This marked a turning point in multimodal generation, further bolstered by the release of CLIP [18], a model trained to align text and images in a shared embedding space. These tools laid the groundwork for the next generation of models, including Google’s Imagen, released in 2022, which used a diffusion-based approach to generate photorealistic images from text [19]. OpenAI followed with DALL-E 2, incorporating CLIP to improve semantic alignment between text and image [20]. Stability AI’s release of Stable Diffusion further democratized access by making high-performing text-to-image Diffusion Models open-source.

Progress continued into 2023, with OpenAI’s DALL-E 3, which introduced improved training methods and data alignment strategies, enhancing the accuracy and consistency of text-to-image synthesis [21]. At the same time, major tech companies integrated generative AI into user-facing products. Microsoft embedded DALL-E-based models into its Designer and Image Creator platforms, making advanced image-generation tools more accessible to non-experts [22]. Meanwhile, Meta developed the Segment Anything Model (SAM) [23], a zero-shot image segmentation model that expanded the toolkit for image manipulation and interactive generation, also enabling new applications in energy sciences [24].

In 2024, text-to-image and image-to-image synthesis models became even more refined. Google advanced its Imagen family with improvements in photorealism and semantic parsing. Meta enhanced its Emu architecture, optimizing for speed and quality and experimenting with hybrid models that combine diffusion processes and VAEs [25,26,27]. OpenAI continued to iterate on the DALL-E line, focusing on higher fidelity and incorporating LLM-based refinements. Anthropic also entered the field, exploring visual generation in conjunction with its Claude model, while Microsoft expanded its ecosystem integrations for generative design tools.

So far, 2025 has continued the trend of Diffusion Models dominating the landscape of image generation. Recent advancements have further enhanced the capabilities of Diffusion Models for both image synthesis [28] and super-resolution [29]. In particular, breakthrough applications have emerged in scientific imaging, for example, models in [30] demonstrated a promising generative approach for dehazing satellite images.

Today, Diffusion Models dominate the generative image landscape, often operating in latent spaces defined by VAEs for greater efficiency. Although GANs remain relevant in niche tasks such as upscaling and style transfer, Diffusion Models now serve as the foundation for most text-to-image and image-to-image systems. A defining trend across recent developments is the integration of large language models (LLMs), which enhance generative fidelity by better interpreting prompts and guiding image synthesis. As these technologies continue to mature, understanding their mathematical foundations, computational requirements, and potential pitfalls is critical, especially when applying them to scientific image generation, where precision and trustworthiness are mandatory.

**Figure 2:**
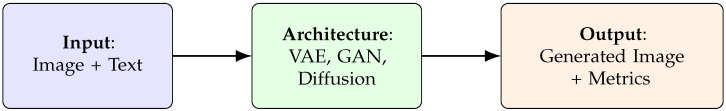
Image-generation pipeline: The Input stage processes a combination of text/prompt and scientific images. Next, a single Architecture (VAE, GAN, or Diffusion) is employed based on this input. Finally, Output assessment can be performed either qualitatively, by visualizing the generated image, or quantitatively, using metrics such as SSIM, LPIPS, FID, and CLIPScore.

This paper describes key generative architectures (Section 3, Section 4 and Section 5), highlighting their underlying mathematical foundations, computational demands, and prevalent challenges in scientific image generation. Section 6 explains the Experimental Setup, including a comparative analysis on the generative methods and a summary on the selected models and APIs for image generation. Subsequently, Section 7 details the metrics for the verification and validation protocols applied to selected models, and discusses the experimental results using each of the selected models against energy-centric scientific data (Figure 2). Finally, Section 8 summarizes the results and discusses capabilities and limitations across datasets and Section 9 draws conclusions about this investigation and future directions.

## 3. Key Generative Architecture: Variational Auto-Encoder (VAE)

First introduced in 2013, the Variational Auto-Encoder (VAE) [31] is a type of generative neural network capable of learning a probability distribution over a set of data points without labels. It learns to encode input data into a lower-dimensional latent space and decode it back to the original image space by sampling latents, while ensuring the latent representations follow a known probability distribution.

A VAE is a latent-variable model with an intractable posterior distribution, which prevents direct likelihood evaluation. Instead, it approximates the posterior using variational inference. This means that the VAE must optimize a lower bound on the likelihood because marginalizing over the latent space is intractable. Intuitively, latent variables (LVs) provide a more compact representation of the data by capturing its underlying structure. More formally, they are the result of transforming data points into a continuous, lower-dimensional space that reveals the essential features of the observed data.

Throughout this paper, we denote vectors using boldface (e.g., x, y, z). In the context of VAEs, let D be the dimensionality of the observed data, then $x\in R^{D}$ represents the observed data (e.g., images), and $z\in R^{d}$ represents a latent variable, where typically *d* is the dimension of each latent variables and d≪D.

Mathematically, given a data point x drawn from an unknown distribution p(x), and a latent variable z from a prior p(z), the following relationships hold:

- p(z) is the prior distribution over LVs;
- $p_{\theta }\left(x\right)$ is the marginal distribution (model goal, intractable to compute directly);
- $p_{\theta }\left(x|z\right)$ is the likelihood or decoder, parametrized by θ, mapping latents z to data points x;
- $p_{\theta }\left(x,z\right)=p_{\theta }\left(x|z\right)p\left(z\right)$ is the joint distribution of data points and latent variables;
- $p_{\theta }\left(z|x\right)$ is the posterior distribution (approximated during training), which describes z that can be produced by x.

**Figure 3:**
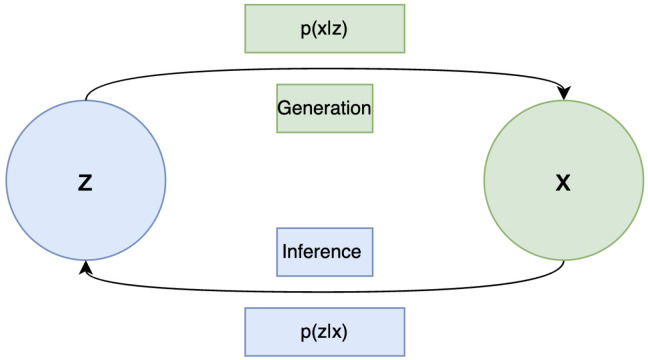
Variational inference and generative process in the VAE.

The generative process in VAEs consists of sampling a latent variable from the prior distribution z∼p(z), then generating a data sample from the conditional distribution $x∼p_{\theta }\left(x|z\right)$. During inference, given a data point x, the posterior $p_{\theta }\left(z|x\right)$ is needed to sample a latent variable z that captures the underlying representation of x (see Figure 3). However, since the true posterior $p_{\theta }\left(z|x\right)$ is intractable, VAEs instead use a variational approximation $q_{\phi }\left(z|x\right)$, parametrized by ϕ, typically implemented as the encoder network.

To find the parameters of the marginal distribution $p_{\theta }\left(x\right)$, we can apply gradient descent, which translates into computing the following (non-tractable gradient):

(1) $$\begin{array}{c} \nabla \log p_{\theta }\left(x\right)=\nabla_{\theta }\log\int p_{\theta }\left(x,z\right)dz=\nabla_{\theta }\log\int p_{\theta }\left(x|z\right)p\left(z\right)dz. \end{array}$$

**Figure 4:**
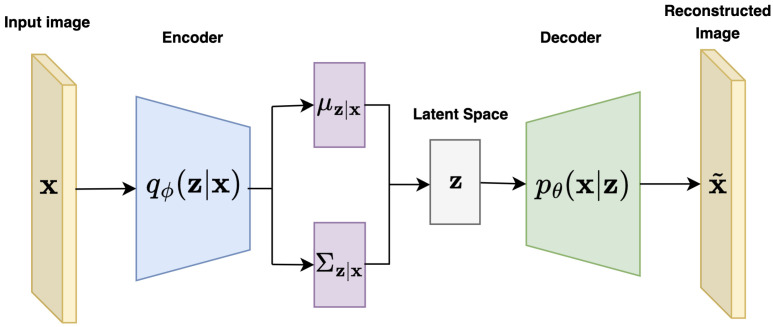
VAE encoder–decoder architecture.

The goal of variational inference is to approximate the intractable posterior distribution $p_{\theta }\left(z|x\right)$ with a tractable explicit distribution $q_{\phi }\left(z|x\right)$, known as variational posterior (see Figure 4, encoder block). Here, the parameters θ represent the parameters of the generative model (decoder), while ϕ corresponds to the parameters of the inference model (encoder). By using this approximation, Bayesian inference can be reformulated as an optimization problem [32]. Specifically, training involves minimizing the Kullback–Leibler (KL) divergence between $q_{\phi }\left(z|x\right)$ and the true posterior $p_{\theta }\left(z|x\right)$, defined as:

(2) $$\mathrm{KL}\left(q_{\phi }\left(z|x\right)∥p_{\theta }\left(z|x\right)\right)=\int q_{\phi }\left(z|x\right)\log\frac{q_{\phi }\left(z|x\right)}{p_{\theta }\left(z|x\right)}dz=E_{z∼q_{\phi }\left(z|x\right)}\left[\log\frac{q_{\phi }\left(z|x\right)}{p_{\theta }\left(z|x\right)}\right].$$

Sampling directly from the variational posterior distribution $q_{\phi }\left(z|x\right)$ is non-differentiable, preventing gradient backpropagation during training. To address this, Kingma and Welling [31] introduced a reparameterization trick, which transforms the sampling step into a differentiable operation. Specifically, instead of directly sampling the distribution parameterized by mean μ and standard deviation σ, the latent variables are obtained by adding parameter-independent noise ϵ drawn from a standard normal distribution, enabling gradient computations:

(3) z=μ+σ·ϵ,

where ϵ∼N(0,1), which is independent of the network parameters. The new z is now a deterministic function of μ, σ, and ϵ. Since μ and σ are outputs of the neural network, we can now backpropagate through them. That way instead of learning z directly, the network learns μ(x) and σ(x) to shape the latent distribution and sampling occurs outside of the computational graph (with ϵ), making it possible to compute gradients and optimize the VAE via gradient descent.

Once the prior distribution is defined, the generative process (decoder) of the VAE consists of the following steps (see Figure 4, decoder block):

- Sample latent variable z∼N(0,I);
- Compute parameters $\mu_{x},\sigma_{x}$ through the decoder network;
- Generate a data point x by sampling from $N(\mu_{x},\sigma_{x}^{2}I)$.

The training objective of the VAE is to maximize the Evidence Lower Bound (ELBO), equivalently formulated as maximize the following loss function:

(4) $$L_{\theta ,\phi }\left(x\right)=E_{q_{\phi }\left(z|x\right)}\left[\log p_{\theta }\left(x|z\right)\right]-\mathrm{KL}\left(q_{\phi }\left(z|x\right)∥p\left(z\right)\right).$$

The first term of this equation, known as the reconstruction error, quantifies how well the decoder reconstructs the input data x from the latent representation z. The second term measures the KL divergence between the variational posterior $q_{\phi }\left(z|x\right)$ and the prior $p_{\theta }\left(z\right)$, encouraging the latent space produced by the encoder to remain regularized, continuous, and consistent with the prior assumptions.

### β-VAE

The *β*-VAE (Beta-Variational Autoencoder) is a modification of the standard Variational Autoencoder (VAE) presented in 2017 [33], and that introduces a weighting adjustable factor β to control the trade-off between reconstruction fidelity and the disentanglement of the learned latent representations. In contrast with standard VAEs, β-VAE modifies this objective by scaling the KL term with a hyperparameter β≥1. When β>1, the model is encouraged to learn more disentangled and factorized latent representations at the cost of some reconstruction accuracy. This is particularly useful in unsupervised learning where interpretability of latent factors is important. The objective function of this model is similar to Equation (4) with the additional β factor:

(5) $$L_{\beta -VAE}\left(x\right)=E_{q_{\phi }\left(z|x\right)}\left[\log p_{\theta }\left(x|z\right)\right]-\beta \mathrm{KL}\left(q_{\phi }\left(z|x\right)∥p\left(z\right)\right).$$

Using β-VAE is particularly beneficial when focusing on learning interpretable, disentangled and structured latent representations. Such representations allow better analysis, manipulation and control of the latent space and independent generative factors (e.g., shape, size, orientation).

## 4. Key Generative Architecture: Generative Adversarial Networks (GANs)

**Figure 5:**
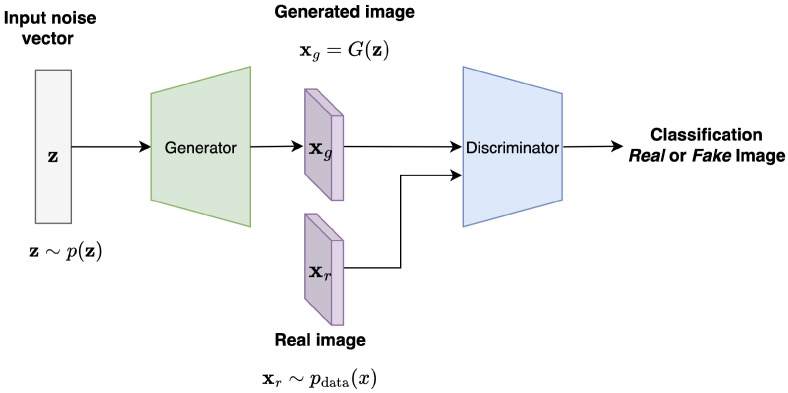
Vanilla GAN architecture, illustrating the generator (taking a noise vector as input) and discriminator (evaluating real and generated images individually).

The Generative Adversarial Network (GAN), introduced in 2014 [34,35], represents a major advance in generative learning. GANs comprise two competing neural networks: a generator and a discriminator, where the generator aims to produce synthetic data, and the discriminator attempts to distinguish between real data and synthetic data (see Figure 5).

Formally, GAN training involves solving a min-max adversarial optimization problem, described as follows:

- The generator G(z) maps random noise z∼p(z) (also called latents and where p(z) is the prior over the latents) to the data distribution $p_{\mathrm{data}}\left(x\right)$ and outputs the synthetic image in the shape of a 1D-vector $x_{g}$. The stochasticity given by this random sampling will provide a non-deterministic output, which is how the model creates diversity in the generation process. The goal here is to fool the discriminator and minimize log(1−D(G(z))), which amounts to maximizing the discriminator’s error in classifying the generated images as fake.
- The discriminator D(x) takes as input a real $x_{r}$ and synthetic image $x_{g}$ (generated by the generator) and outputs the probability that the image x comes from the real data distribution or not. The goal here is to maximize the loss function or the probability that it correctly classifies real and fake images.

This adversarial process drives both the generator and the discriminator to improve, resulting in high-quality synthetic data. In addition, the fact that the generator is only trained to fool the discriminator makes this Vanilla GAN model unsupervised. The goal of the GAN is to solve the min-max game or adversarial game between the generator and the discriminator with the following objective function and optimization problem:

(6) $$\min_{G}\max_{D}V\left(G,D\right)=E_{x∼p_{\mathrm{data}}\left(x\right)}\left[\log D\left(x\right)\right]+E_{x∼p(z)}\left[\log\left(1-D\left(G\left(z\right)\right)\right)\right],$$

where D(x) is the probability that x is real, G(z) is the generated sample, and thus D(G(z)) is the probability that the generated image given latent z is real.

One of the most common limitations of GANs is the so-called **mode collapse** problem where the generator fails to accurately represent the pixel space of all possible outputs. This issue is common in high-resolution images, where too many fine-scale features must be captured. In that case, the generator gets stuck in a parameter setting with a similar level of noise that can consistently fool the discriminator and only captures a subset of the real data distribution. It then fails to produce diversity in its outputs and collapses to producing only a few types of synthetic samples.

### 4.1. Conditional GAN (CGAN)

**Figure 6:**
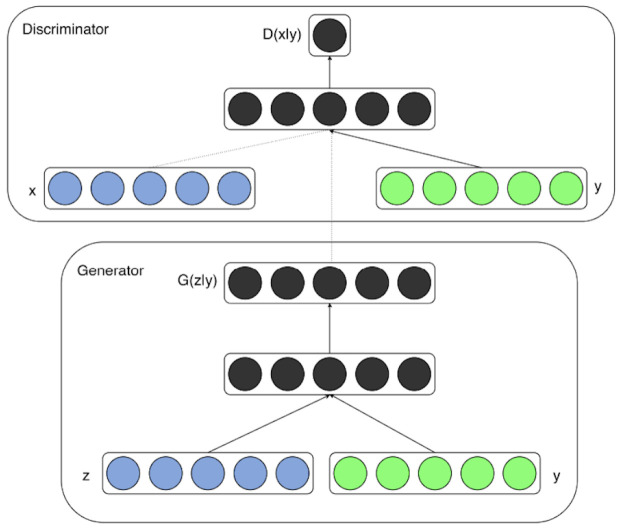
Architecture of the Conditional GAN, where the vector in *green* is associated to the conditional or label vector. Source: [36].

As an extension of the Vanilla GAN, the Conditional GAN was introduced in 2014 [36], and uses conditional information (image or text) to guide the generation process. The CGAN performs conditioning generation by feeding information to both the generator and the discriminator (see Figure 6).

The generator G(z,y) takes as input random noise z, and the conditional embedding y and learns to generate data given this condition, whereas the discriminator D(x,y) learns to classify real and fake images by checking that condition y is met. The updated conditional min-max optimization function becomes:

(7) $$\min_{G}\max_{D}V\left(G,D\right)=E_{x∼p_{\mathrm{data}}\left(x|y\right)}\left[\log D\left(x,y\right)\right]+E_{z∼p(z)}\left[\log\left(1-D\left(G\left(z,y\right),y\right)\right)\right].$$

### 4.2. Deep Convolutional GAN (DCGAN)

**Figure 7:**
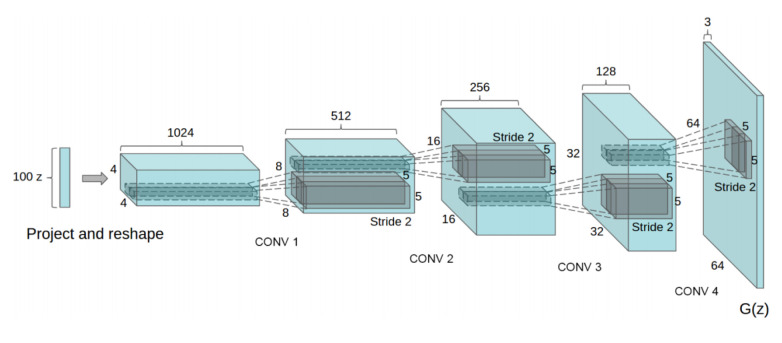
Architecture of the generator block of the DCGAN model, composed of convolutional blocks and taking as input a latent vector and outputs a synthetic image. Source: [37].

Following the initial development of GANs, various architectures emerged, notably Deep Convolutional Generative Adversarial Networks (DCGANs) introduced by Radford et al. in 2015 [37], which extended the foundational GAN framework. While the Vanilla GAN architecture contains downsampling and upsampling layers with ReLU activations and a sigmoid activation for the discriminator, this variant of the GAN is made of strided convolution layers in both the Discriminator and the Generator (as illustrated in Figure 7), along with batch normalization layers, and LeakyReLU activation functions. This architecture is adapted to small-size images such as RGB inputs of shape *(3,64,64)* and struggles with high-resolution images.

### 4.3. Architectural Innovations Derived from CGAN and DCGAN

#### 4.3.1. Pix2Pix

Pix2Pix is a type of Conditional GAN framework introduced in 2017 by Isola et al. [10], which learns a mapping from an input image x and random noise vector z to a target image y, using a U-Net [38]-based generator *G* and a convolution-based discriminator *D* (called PatchGAN). The adversarial loss encourages the generator to produce outputs that are indistinguishable from real images, conditioned on the input:

(8) $$L_{\mathrm{cGAN}}\left(G,D\right)=E_{x,y∼p_{\mathrm{data}}\left(x,y\right)}\left[\log D\left(x,y\right)\right]+E_{x∼p_{\mathrm{data}}\left(x\right),z∼p\left(z\right)}\left[\log\left(1-D\left(x,G\left(x,z\right)\right)\right)\right].$$

In addition to the adversarial loss, Pix2Pix introduces a reconstruction loss based on the $L_{1}$ distance between the generated image and the ground truth, which encourages the generator to produce images that are structurally close to the target:

(9) $$E_{x,y∼p_{\mathrm{data}}\left(x,y\right)}[{∥y-G\left(x,z\right)∥}_{1}].$$

The total objective for the generator combines both losses:

(10) $$L_{\mathrm{Pix2Pix}}\left(G,D\right)=L_{\mathrm{cGAN}}\left(G,D\right)+\lambda \;E_{x,y∼p_{\mathrm{data}}\left(x,y\right)}[{∥y-G\left(x,z\right)∥}_{1}],$$

where λ is a hyperparameter that controls the relative importance of the $L_{1}$ loss.

#### 4.3.2. CycleGAN

CycleGAN is a generative model designed in 2017 [11], for unpaired image-to-image translation. It learns to translate images from one domain *X* (e.g., horses) to another domain *Y* (e.g., zebras) without requiring paired training examples.

**Figure 8:**
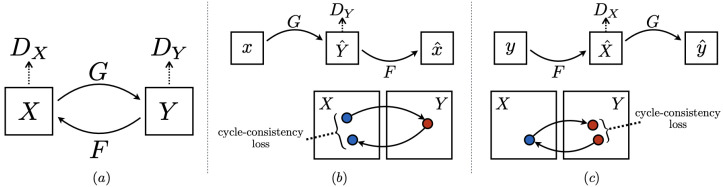
(**a**) CycleGAN architecture containing two mapping functions F and G and two associated adversarial discriminators $D_{Y}$ and $D_{X}$. (**b**) Forward cycle-consistency loss: x→G(x)→F(G(x))≈x. (**c**) Backward cycle-consistency loss: y→F(y)→G(F(y))≈y, where blue dots referring to outputs of domain X and red dots referring to outputs of domain Y. Source: [11].

The model consists of two generators and two discriminators (see Figure 8) and is defined by the following structure:

- Generator G:X→Y;
- Generator F:Y→X;
- Discriminator $D_{Y}$: distinguishes real *Y* images from generated ones G(x);
- Discriminator $D_{X}$: distinguishes real *X* images from generated ones F(y).

Each generator is trained with a standard GAN loss.

The **full objective** combines both adversarial and cycle consistency losses:

(11) $$L\left(G,F,D_{X},D_{Y}\right)=L_{\mathrm{GAN}}\left(G,D_{Y},X,Y\right)+L_{\mathrm{GAN}}\left(F,D_{X},Y,X\right)+\lambda L_{\mathrm{cyc}}\left(G,F\right),$$

where λ is a hyperparameter controlling the importance of the cycle consistency loss. CycleGAN thus enables high-quality, unpaired image-to-image translation through adversarial learning and cyclic reconstruction. For more details on the explicit loss formulation, see Appendix A.

#### 4.3.3. StyleGAN

StyleGAN (Style-Based Generative Adversarial Network) is a type of GAN that was introduced by NVIDIA in 2018 [12] and was initially applied to image face synthesis. It relies on style-based architecture with convolutional layers. This new GAN architecture allows control over different aspects of the image by learning high-level features without supervision and stochastic variation (random noise) in the synthetic images (fine-scale attributes such as eyes and hair).

**Figure 9:**
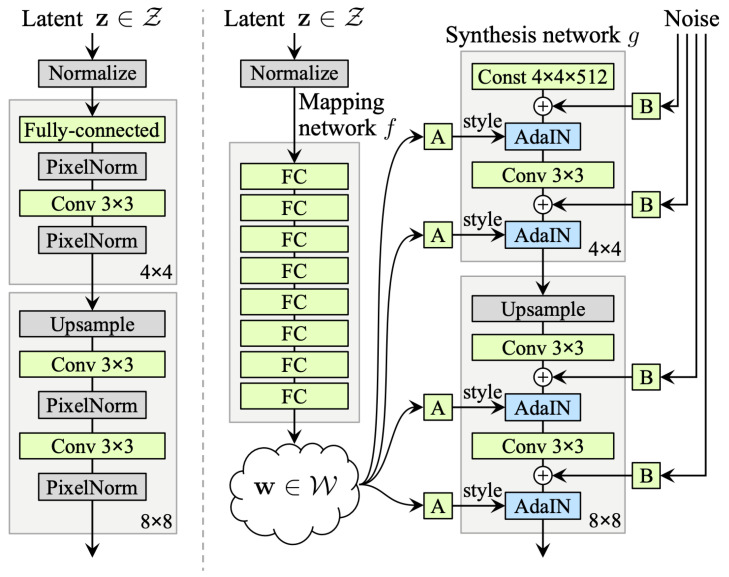
(**Left**) Traditional generator architecture takes a noise vector z as input, a (**right**) style-based generator with an additional mapping network *f* and an intermediate latent space *W* that controls the generator through AdaIN at each convolution layer. w∈W is added through a learned affine transform “A”. Gaussian noise is added after each convolution, before evaluating the nonlinearity through “B”, which applies learned per-channel scaling factors to the noise input. Source: [12].

Let z be a latent variable, input of the generator. Then as illustrated on the right side of Figure 9, z is mapped through multiple fully connected (FC) layers and outputs a vector w which will be an intermediate representation fed to each convolutional block, through Adaptive Instance Normalization (AdaIN) that normalizes feature maps separately. Gaussian noise is then added to the feature maps after each convolutional layer, which allows conservation of global aspects.

The StyleGAN generator architecture enables precise control over image synthesis through scale-specific style modifications.

By leveraging a mapping network and affine transformations, the model samples styles from a learned distribution, while the synthesis network constructs an image by integrating these styles. Crucially, each style’s influence remains localized within the network and adjusting a subset of styles primarily alters corresponding aspects of the image. This localization effect arises from the AdaIN mechanism. Before applying style-based transformations, AdaIN standardizes each channel by enforcing a zero mean and unit variance. Only afterward does it introduce new scales and biases dictated by the style parameters. Because this process eliminates dependence on the original feature statistics, each style exclusively governs a single convolution before the next AdaIN operation takes over. This sequential modulation allows for fine-grained and independent control over different levels of the generated image.

The discriminator in StyleGAN is a standard Convolutional Neural Network (CNN) designed to distinguish between real and generated images.

#### 4.3.4. GigaGAN

GANs were the traditional choice for text-to-image generation until the release of large Diffusion Models such as DALL-E, Imagen, and Stable Diffusion. These new Diffusion Models have parameters on the scale of billions, while the SOTA GAN model had only 75 million parameters. The disparity in image quality was attributed to the massive gap in parameter counts. GigaGAN was able to create a scalable architecture that far exceeds the size of previous GAN models and has competitive performance to Diffusion Models while being orders of magnitude faster [39]. GigaGAN is a private model whose results were published by Adobe in 2023. While there is a community implementation [40] of the work presented in [39], there have been no other attempts to make a publicly available version of this model to test at the time of writing this paper. Although the results of the paper are promising, training the model from scratch to recreate the capabilities and testing on scientific images are beyond the scope of this paper.

#### 4.3.5. Other GAN-Based Models

StackGAN [41] and Attention GAN (AttnGAN) [42] are notable Conditional GAN architectures that have significantly advanced the field of conditional image generation. StackGAN introduced a hierarchical approach, generating low-resolution images and iteratively refining them to high-resolution outputs. AttnGAN innovated with attention mechanisms (see explanation of **attention mechanisms** in Section 5.2.1), allowing the model to selectively attend to specific words or phrases in the text description when generating the corresponding image regions. Progressive GAN [43] and BigGAN [44] are two other influential models that significantly advanced image generation. Progressive GAN grows the generator and discriminator networks gradually, from low to high resolution, starting with tiny images (e.g., 4 × 4) and incrementally adding layers to reach resolutions such as 1024 × 1024. This strategy stabilizes training and allows the model to learn coarse features before fine details. In contrast, BigGAN focuses on scaling up model size and dataset complexity. It introduces class-conditional generation with large batch sizes and deep architectures, enabling the production of high-fidelity, diverse images across 1000 ImageNet categories. BigGAN employs techniques such as the truncation trick to balance the trade-off between image quality and diversity. In this method, instead of sampling noise vectors *z* from the full standard normal distribution z∼N(0,1), the samples are clipped or resampled to lie within a certain range closer to the mean. This limits extreme values, which tends to improve image fidelity at the cost of reduced variability.

Table 1 summarizes the discussion about GAN models, showcasing their diverse architectures and applications, from early convolutional models like DCGAN to modern style-based approaches such as StyleGAN and GigaGAN. While early models focused on simple image generation and translation, later advancements incorporated techniques like cycle consistency (CycleGAN), attention mechanisms (AttnGAN), and multi-stage refinement (StackGAN) to improve conditional generation and overcome common limitations like mode collapse and low resolution.

## 5. Key Generative Architecture: Diffusion Models

In thermodynamics, diffusion refers to the spontaneous flow of particles from regions of high concentration to regions of low concentration, ultimately moving the system toward a state of equilibrium. In statistics, the concept of diffusion draws a similar analogy: it describes the process of transforming a complex data distribution $p_{\mathrm{complex}}$ into a simpler, predefined distribution $p_{\mathrm{prior}}$ over the same domain. Formally, this is achieved via a first transformation τ such that:

(12) $$x_{0}∼p_{\mathrm{complex}}\Rightarrow \tau \left(x_{0}\right)∼p_{\mathrm{prior}}.$$

Describing this analogy in terms of entropy provides additional insight: both in thermodynamics and in statistics, diffusion involves an increase in disorder or uncertainty in the forward direction. That is, a structured, high-information (low-entropy) distribution is progressively mapped to an unstructured, high-entropy distribution (e.g., isotropic Gaussian noise).

The second transformation reverses this stochastic process, reducing entropy and gradually transforming samples from the simple distribution $p_{\mathrm{prior}}$ back into samples from $p_{\mathrm{complex}}$. These two mappings constitute the *forward process* (diffusion) and the *reverse process* (denoising generation), which together form the foundation of diffusion-based generative models.

**Figure 10:**
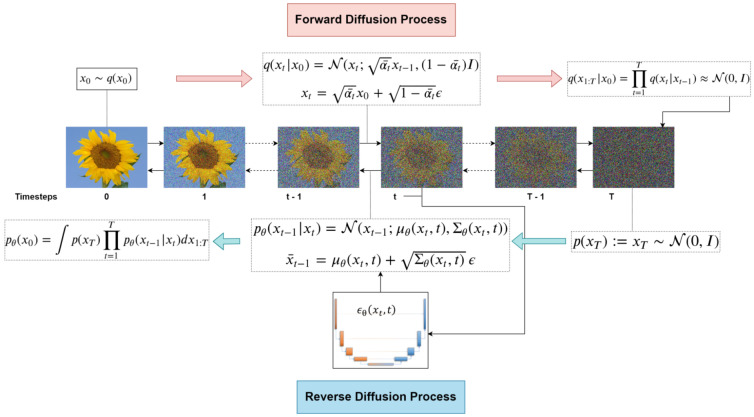
Diffusion model based on DDPMs: (**Top**) forward process; (**Bottom**) reverse process. Source: [45].

Diffusion Models, now producing SOTA high-fidelity and diverse images, have evolved from the initial work of Sohl-Dickstein et al. in 2015 [15], to the significantly impactful Denoising Diffusion Probabilistic Models (DDPM) by Ho et al. in 2020 [16]. Diffusion Models differ from previous generative models as they decompose the image-generation process through small denoising steps. They take an input image $x_{0}$ and gradually add Gaussian noise (forward process). The second part of the network (reverse process or sampling process), consists of removing the noise to obtain new data (see Figure 10).

The *forward* process consists of a Markov chain of *T* steps, where Gaussian noise is incrementally added to an input image $x_{0}∼q\left(x_{0}\right)$ to produce noisy latent variables $x_{1},\ldots ,x_{T}$ of the same dimensionality. The variance of added noise is controlled by a schedule $\beta_{1},\ldots ,\beta_{T}$, often linear or cosine.

During training, a neural network approximates the reverse transitions $p_{\theta }\left(x_{t-1}|x_{t}\right)$ using Gaussian parameterization. The objective is to minimize the negative log-likelihood, which is estimated via the Evidence Lower Bound (ELBO):

(13) $$\begin{array}{c} \log p(x)\geq E_{q(x_{1}|x_{0})}\left[\log p_{\theta }\left(x_{0}|x_{1}\right)\right] \end{array}$$

(14) $$\begin{array}{cc} \; & \;-D_{KL}\left(q\left(x_{T}|x_{0}\right)∥p\left(x_{T}\right)\right) \end{array}$$

(15) $$\begin{array}{cc} \; & \;-\sum_{t=2}^{T}E_{q(x_{t}|x_{0})}\left[D_{KL}\left(q\left(x_{t-1}|x_{t},x_{0}\right)∥p_{\theta }\left(x_{t-1}|x_{t}\right)\right)\right] \end{array}$$

(16) $$\begin{array}{cc} \log p(x) & \geq L_{0}-L_{T}-\sum_{t=2}^{T}L_{t-1}, \end{array}$$

where:

- $L_{0}=E_{q(x_{1}|x_{0})}\left[\log p_{\theta }\left(x_{0}|x_{1}\right)\right]$ is the reconstruction term;
- $L_{T}=D_{KL}\left(q\left(x_{T}|x_{0}\right)∥p\left(x_{T}\right)\right)$ quantifies how close the noisy latent $x_{T}$ is to a standard Gaussian;
- $\sum_{t=2}^{T}L_{t-1}$ measures the gap between the true reverse process and the learned denoising model.

The reverse model is typically implemented as a U-Net conditioned on timestep embeddings and trained using a mean squared error loss between the true and predicted noise. A simplified version of the training loss, used in DDPM training, is derived that enables the model to predict the noise ϵ added at each timestep *t*, rather than directly reconstructing $x_{0}$.

(17) $$\begin{array}{cc} L_{\mathrm{simple}}\left(\theta \right) & =E_{t,x_{0},\epsilon }[∥\epsilon -\epsilon_{\theta }\left(\sqrt{{\bar{\alpha }}_{t}}x_{0}+\sqrt{1-{\bar{\alpha }}_{t}}\epsilon ,t\right){∥}^{2}]. \end{array}$$

For a complete derivation of the forward and reverse processes, we refer the reader to Appendix B.

The model is typically implemented using a U-Net with residual blocks, group normalization, and self-attention. The timestep *t* is embedded (e.g., using a cosine embedding) and injected into each residual block.

### 5.1. Score-Based Generative Models

**Figure 11:**
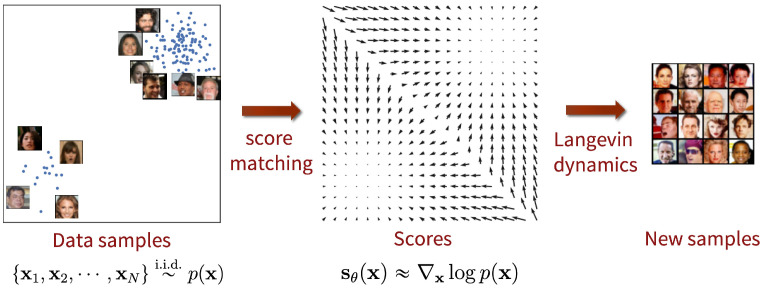
Score-based generative modeling with score matching and Langevin dynamics. Source: [47].

Score-Based Diffusion Models (SBDMs) are a class of Diffusion Models proposed by [46,47] that combine score functions (gradient of the log probability density function) and Langevin dynamics (iterative process where we draw samples from a distribution based only on its score function, as illustrated in Figure 11). The gradient of the log probability density function, also called the score function, is the mathematical tool that allows generative models to transform random noise into realistic data by following the estimated directions where the data probability density grows most.

This approach builds on the principle of score-modeling and score-matching [48], enabling the training of deep neural networks to approximate the score of complex, high-dimensional data distributions. Unlike methods such as Variational Autoencoders (VAEs), which require a tractable normalizing constant, or Generative Adversarial Networks (GANs), which rely on adversarial training, score-based modeling bypasses both constraints. Instead of modeling the probability density function p(x) directly, a neural network $s_{\theta }$ is trained to approximate its score function $\nabla_{x}\log p\left(x\right)$ by minimizing the following training objective:

(18) $$E_{p_{x}}[∥\nabla_{x}\log p(x)-s_{\theta }(x){∥}_{2}^{2}]=\int (p\left(x\right)∥\nabla_{x}\log p\left(x\right)-s_{\theta }\left(x\right){∥}_{2}^{2})dx.$$

Once the score-based model is trained and $s_{\theta }$ is obtained, the next step consists in generating samples using a Langevin Dynamics Markov chain Monte Carlo (MCMC) procedure by starting from an arbitrary prior distribution and iterating the following update (for i=1,…,K):

(19) $$x_{i+1}\leftarrow x_{i}+\epsilon \nabla_{x}\log p\left(x_{i}\right)+\sqrt{2\epsilon }\;z_{i},$$

where $z_{k}∼N\left(0,I\right)$. When the step size ϵ→0 and the number of iterations K→∞, the distribution of $x_{k}$ obtained from this procedure converges to a sample from p(x) under some regularity conditions. In practice, the error is negligible when ϵ is sufficiently small and *K* is sufficiently large.

#### 5.1.1. Noise Conditional Score Networks (NCSN)

While Langevin dynamics can sample p(x) using the approximated score function, directly estimating $\nabla_{x}\log p\left(x\right)$ is difficult and imprecise: the estimated score functions are usually inaccurate in low-density regions, where few data points are available and as a result, the quality of the data sampled using Langevin dynamics is poor. To address this, one solution consists in learning score functions at various noise levels, which can be achieved by perturbing the data with multiple scales of Gaussian noise [46]. Therefore, given the data distribution p(x), we perturb it with Gaussian noise $N(0,\sigma_{i}^{2}I)$ where i=1,2,…,L and $\sigma_{1}<\sigma_{2}<\ldots <\sigma_{L}$ to obtain a noise-perturbed distribution:

(20) $$p_{\sigma_{i}}\left(x\right)=\int p\left(y\right)N\left(x;y,\sigma_{i}^{2}I\right)\;dy,$$

which we can draw samples from by sampling x∼p(x) and computing $x+\sigma_{i}z$, where z∼N(0,I). Finally we train a network $s_{\theta }\left(x,i\right)$, known as the Noise Conditional Score-Based Network (NCSN), to estimate the score function $\nabla_{x}\log p_{\sigma_{i}}\left(x\right)$. The training objective is a weighted sum of Fisher divergences for all noise levels:

(21) $$\sum_{i=1}^{L}\lambda \left(i\right)\;E_{p_{\sigma_{i}}\left(x\right)}\left[{∥\nabla_{x}\log p_{\sigma_{i}}\left(x\right)-s_{\theta }\left(x,i\right)∥}_{2}^{2}\right].$$

Similarly to the previous section, once we obtain $s_{\theta }\left(x,i\right)$, we can apply the Langevin Dynamics MCMC procedure to sample new data points.

#### 5.1.2. Score-Based Diffusion Through Stochastic Differential Equations (SDE)

**Figure 12:**
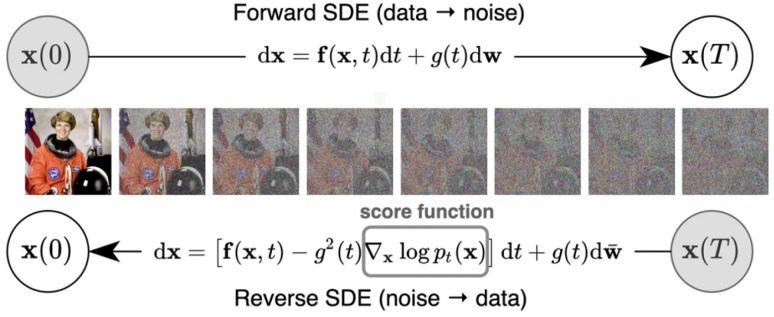
Score-based generative modeling through SDE by transforming input data to a noise distribution through a continuous-time SDE and reversing the process using the score function of the distribution at each intermediate time step. Source: [47].

Song et al. [46,47] unify Noise Conditional Score Networks (NCSNs) and Denoising Diffusion Probabilistic Models (DDPMs) by introducing a continuous-time generative model based on stochastic differential equations (SDEs). In contrast to perturbing data with a discrete set of noise levels, they define a continuous-time diffusion process ${\left\{x\left(t\right)\right\}}_{t\in [0,T]}$, which gradually transforms data into a tractable noise distribution. This forward process, going from an input image x(0) to random noise x(T) as in Figure 12, is governed by a fixed reversible SDE with no learnable parameters:

(22) dx=f(x,t)dt+g(t)dw,

where:

- $x\left(t\right)\in R^{d}$ is the state at time *t*;
- $f:R^{d}\times \left[0,T\right]\to R^{d}$ is a vector valued function called the drift function, and f(·) is always of the form f(x,t)=f(t)x;
- $g:\left[0,T\right]\to R_{+}$ is a real-valued function corresponding to the diffusion coefficient;
- dw is a standard Wiener process (Brownian motion with infinitesimal white noise).

To determine the specific forms of f(t) and g(t), two commonly used noise schedules are presented in [47] (and originally in [16,46]). A full derivation of these formulations can be found in Appendix C.

Let x denote x(t), i.e., the state of the process at time *t*, and $x\left(0\right)=x_{0}$. The marginal distribution $p_{t}\left(x\right)$ is then obtained by integrating the perturbation kernels over $x_{0}$ (Equation (13) of [49]):

(23) $$p_{t}\left(x\right)=\int_{R^{d}}p_{0t}\left(x|x_{0}\right)\;p_{\mathrm{data}}\left(x_{0}\right)\;dx_{0},$$

where $p_{0t}\left(x\left(t\right)|x\left(0\right)\right)$ is Gaussian and defined in Appendix C.

The next step consists in learning a time-dependent score function $s_{\theta }\left(x,t\right)\approx \nabla_{x}\log p_{t}\left(x\right)$. Since the true score function, $\nabla_{x}\log p_{t}\left(x\right)$ is intractable, it is approximated using the score neural network $s_{\theta }\left(x,t\right)$. Then, using the identity:

(24) $$\nabla_{x}\log p_{t}\left(x\right)=E_{p_{0t}\left(x_{0}|x\right)}\left[\nabla_{x}\log p_{0t}\left(x|x_{0}\right)\right],$$

we train the score network $s_{\theta }\left(x,t\right)$ to match $\nabla_{x}\log p_{0t}\left(x|x_{0}\right)$ on average over samples $x∼p_{0t}\left(x|x_{0}\right)$.

This gives rise to training the model by minimizing the Continuous-Time Weighted Score-Matching Loss:

(25) $$L\left(\theta \right)=E_{t∼U(0,T)}[\lambda \left(t\right)E_{x_{0}∼p_{\mathrm{data}},\;x∼p_{0t}\left(x|x_{0}\right)}\left[{∥\nabla_{x}\log p_{0t}\left(x|x_{0}\right)-s_{\theta }\left(x,t\right)∥}_{2}^{2}\right],$$

where:

- $s_{\theta }\left(x,t\right)$ is the score network approximating $\nabla_{x}\log p_{t}\left(x\right)$;
- $p_{t|0}\left(x|x_{0}\right)$ is the transition kernel of the forward SDE;
- λ(t) is a weighting function over time (often noise-dependent);
- U(0,T) is the uniform distribution over the time interval [0,T].

Once the score-based model is trained, it is possible to generate new samples by computing the well-defined reverse SDE, under appropriate regularity conditions (e.g., those from Anderson’s theorem [50]):

(26) $$dx=\left[f\left(x,t\right)-g^{2}\left(t\right)\nabla_{x}\log p_{t}\left(x\right)\right]\;dt+g\left(t\right)\;d\bar{w},$$

where $\bar{w}$ is a standard Wiener process evolving backward in time, and $p_{t}\left(x\right)$ denotes the marginal density of x. Since $\nabla_{x}\log p_{t}\left(x\right)$ is not analytically available, it is learned via a time-dependent score network $s_{\theta }\left(x,t\right)\approx \nabla_{x}\log p_{t}\left(x\right)$.

The Euler–Maruyama method is the default solver used in early works, such as Song et al. [47], where it is employed to approximate the reverse-time SDE during the sampling process. Subsequent improvements led to the adoption of higher-order solvers. For instance, Karras et al. [49] explore the design space of noise schedules and use Heun’s method, a second-order stochastic Runge–Kutta scheme, for improved sampling quality.

Song et al. [47] also propose the probability flow ODE which shares the same marginal distributions $p_{t}\left(x\right)$ as the corresponding SDE:

(27) $$dx=f\left(x,t\right)-\frac{1}{2}g^{2}\left(t\right)\nabla_{x}\log p_{t}\left(x\right)dt.$$

This formulation allows deterministic sampling from the generative model using numerical ODE solvers. Ultimately, the choice between VP and VE formulations depends on the modeling objective: VP provides controlled noise injection allowing likelihood estimation and discrete-time training, while VE supports direct score-based generation from unbounded priors with flexible noise scales.

Alternatives to score-based modeling, such as flow matching [51], propose training neural fields to match velocity fields derived from optimal transport, providing another pathway for continuous-time generative modeling. Compared to autoregressive models, which generate data sequentially and have shown promise in scalable image synthesis [52], score-based and flow-based approaches enable parallel sampling and have opened new avenues for efficient and high-fidelity generation.

#### 5.1.3. Conditional Image Generation with Guided Diffusion and Classifier Guidance

Similarly to the CGAN, an important extension of the Diffusion Model is the *Guided diffusion* model that enables conditional image generation via classifier gradients. It was introduced by Dhariwal et al. [53] when looking for a way to trade off diversity for image fidelity and were inspired by class-conditional generative models that rely on class label conditioning. In the paper [53], the model adds conditioning information y at each diffusion step: Dhariwal et al. train a separate classifier $p_{\phi }\left(y|x_{t},t\right)$ on noisy images at timestep *t* denoted by $x_{t}$, and then use gradients $\nabla_{x_{t}}p_{\phi }\left(y|x_{t},t\right)$ to guide the diffusion sampling process towards an arbitrary class label y. As discussed in the previous section, score-based Diffusion Models generate samples by predicting the score function $\nabla_{x}\log p\left(x|y\right)$ of the target distribution.

Let us first define $\nabla_{x}\log p\left(x|y\right)$ using Bayes rules and gradient computations:

(28) $$\begin{array}{cc} p(x|y) & =\frac{p(y|x)p(x)}{p(y)}, \end{array}$$

(29) $$\begin{array}{cc} \Rightarrow \nabla_{x}\log p\left(x|y\right) & =\nabla_{x}\log p\left(x\right)+\nabla_{x}\log p\left(y|x\right). \end{array}$$

Then, by adding a guidance weight term *s* to the classifier score term $\nabla_{x}\log p\left(y|x\right)$ to control the sharpness of the distribution (closeness to label y in the generation process), they define a new guided conditional score $\nabla_{x}\log p^{\prime }\left(x|y\right)$ using the previous formulation at each timestep t:

(30) $$\begin{array}{c} \nabla_{x_{t}}\log p_{\theta }^{\prime }\left(x_{t}|y\right)=\nabla_{x_{t}}\log p_{\theta }\left(x_{t}\right)+s\nabla_{x_{t}}\log p_{\phi }\left(y|x_{t}\right), \end{array}$$

where:

- $\nabla_{x_{t}}\log p_{\theta }\left(x_{t}\right)$ is the standard diffusion score;
- $\nabla_{x_{t}}\log p_{\phi }\left(y|x_{t}\right)$ is the classifier guidance term;
- *s* is a scaling coefficient controlling the strength of guidance.

Based on the original mean $\mu_{\theta }\left(x_{t}|y\right)$ and variance $\Sigma_{\theta }\left(x_{t}|y\right)$, classifier guidance modifies the mean to:

(31) $$\hat{\mu }\left(x_{t}|y\right)=\mu_{\theta }\left(x_{t}|y\right)+s\;\Sigma_{\theta }\left(x_{t}|y\right)\nabla_{x_{t}}\log p_{\Phi }\left(y|x_{t},t\right),$$

At each reverse diffusion step *t*, sampling is performed using the perturbed mean $\hat{\mu }\left(x_{t}|y\right)$ and the covariance $\Sigma_{\theta }\left(x_{t}|y\right)$:

(32) $$x_{t-1}∼N\left(\hat{\mu }\left(x_{t}|y\right),\;\Sigma_{\theta }\left(x_{t}|y\right)\right).$$

This formulation explicitly uses both the Diffusion Model’s learned dynamics and the classifier’s gradient signal to steer the sampling process toward samples that are more likely to belong to class y (Algorithm 1, source: [53]).
**Algorithm 1** Classifier guided diffusion sampling, given a diffusion model $\left(\mu_{\theta }\left(x_{t}\right),\Sigma_{\theta }\left(x_{t}\right)\right)$, classifier $p_{\phi }\left(y|x_{t}\right)$, and gradient scale *s*1:**Input:** class label *y*, gradient scale *s*2:$x_{T}\leftarrow$ sample from N(0,I)3:**for** all *t* from *T* to 1 **do**4:   $\mu ,\Sigma \leftarrow \mu_{\theta }\left(x_{t}\right),\Sigma_{\theta }\left(x_{t}\right)$5:   $x_{t-1}\leftarrow$ sample from $N(\mu +s\Sigma \nabla_{x_{t}}\log p_{\phi }\left(y|x_{t}\right),\Sigma )$6:**end for**7:**return** $x_{0}$

The intuition behind this approach is the following:

- If the classifier assigns a high probability to class y for a given noisy image $x_{t}$, it means $x_{t}$ is on the right track.
- If the classifier assigns a low probability, the guidance term nudges $x_{t}$ in a direction that increases $p(y|x_{t})$, pushing the sample towards a more likely image.

We can underline that higher guidance weights *s* enforce more alignment with classifier predictions but may reduce diversity, whereas a lower guidance weights allows more diversity but might not enforce class constraints strongly.

Classifier guidance is commonly used in models such as GLIDE [54] and Imagen [55], making text-to-image generation more controllable.

#### 5.1.4. Conditional Image Generation with Guided Diffusion and Classifier Free-Guidance

Classifier-free guidance, proposed by Ho et al. [56], allows for enhanced control in Diffusion Models by eliminating the need for separate classifiers. Instead of relying on a separate classifier, which increases training complexity and introduces potential bias, classifier-free guidance trains the Diffusion Model to directly learn and combine conditional and unconditional distributions during inference, streamlining the process. In other words, the authors train a conditional Diffusion Model $p_{\theta }\left(x_{t}|y\right)$ and an unconditional model $p_{\theta }\left(x_{t}|y=0\right)$ as a single neural network. Based on Equations (29) and (30), classifier-free guidance linearly combines the score estimates of conditional and unconditional models, which leads to the following formula:

(33) $$\nabla_{x_{t}}\log{\hat{p}}_{\theta }\left(x_{t}|y\right)=\nabla_{x_{t}}\log p_{\theta }\left(x_{t}|0\right)+s\left(\nabla_{x_{t}}\log p_{\theta }\left(x_{t}|y\right)-\nabla_{x_{t}}\log p_{\theta }\left(x_{t}|0\right)\right)$$

This approach is advantageous compared to the previous one as it trains a single model to guide the diffusion process and can take different types of conditional data such as text embeddings. We will see that many models rely on classifier free-guidance especially when training on multimodal data.

### 5.2. Stable Diffusion

#### 5.2.1. Attention Mechanisms

Attention is based on the idea that we should look at all the different words of a sequence at the same time and learn to *pay attention* to the correct ones depending on the task in which we are interested. Attention mechanisms, introduced by Vaswani et al. in [13], can be defined as attention of the same sequence, where, instead of looking for an input–ouput sequence association, we look for probability scores between the elements of the sequence.

The attention mechanism computes a weighted representation of a set of values $V\in R^{n\times d_{v}}$ based on a set of queries $Q\in R^{n\times d_{k}}$ and keys $K\in R^{n\times d_{k}}$, where *n* is the sequence length, $d_{k}$ is the key and query dimensionality, and $d_{v}$ is the value dimensionality.

**Self-Attention.** Self-attention is a special case where the queries, keys, and values come from the same sequence. The scaled dot-product attention is defined as:

(34) $$\mathrm{Attention}\left(Q,K,V\right)=\mathrm{softmax}\left(\frac{QK^{⊤}}{\sqrt{d_{k}}}\right)V,$$

where:

- $Q,K,V\in R^{n\times d_{k}}$ are the query, key, and value matrices, respectively;
- $d_{k}$ is the dimensionality of each query and key vector;
- The dot product $QK^{⊤}\in R^{n\times n}$ produces pairwise similarity scores between all tokens in the sequence.
- The softmax operation normalizes each row to a probability distribution over keys such that for a vector $z={\left\{z_{i}\right\}}_{i\in [1,N]}$, then:
  $$\mathrm{softmax}{\left(z\right)}_{i}=\frac{\exp(z_{i})}{\sum_{j=1}^{n}\exp\left(z_{j}\right)}.$$

This mechanism allows each token to attend to all other tokens, including itself, weighted by their learned importance (a token is a vector representation of a discrete input unit: in NLP, tokens represent words or sub-words whereas in vision models, an image is split into patches, each flattened and projected into a vector which form a sequence input to vision transformer models).

**Multi-head Attention.** Instead of computing attention once, multi-head attention projects the queries, keys, and values *h* times using learnable weight matrices and computes attention in parallel across *h* different heads. For each head i∈{1,…,h}:

(35) $${\mathrm{head}}_{i}=\mathrm{Attention}\left(QW_{i}^{Q},KW_{i}^{K},VW_{i}^{V}\right),$$

where:

- $W_{i}^{Q}\in R^{d_{\mathrm{model}}\times d_{k}}$, $W_{i}^{K}\in R^{d_{\mathrm{model}}\times d_{k}}$, $W_{i}^{V}\in R^{d_{\mathrm{model}}\times d_{v}}$ are learnable projection matrices for the *i*-th head;
- Typically, $d_{k}=d_{v}=d_{\mathrm{model}}/h$, so the concatenation of *h* heads gives the original embedding size.

The outputs of all heads are concatenated and projected through another learnable matrix $W^{O}\in R^{d_{\mathrm{model}}\times d_{\mathrm{model}}}$:

(36) $$\mathrm{MultiHead}\left(Q,K,V\right)=\mathrm{Concat}\left({\mathrm{head}}_{1},\ldots ,{\mathrm{head}}_{h}\right)W^{O},$$

where *Concat* denotes the concatenation of the outputs from the *h* individual attention heads along the feature dimension, resulting in a single tensor of shape $R^{T\times (h\cdot d_{k})}$, where *T* is the sequence length and $d_{k}$ is the dimensionality of each head’s output. This concatenated tensor is then linearly projected back to $R^{T\times d_{\mathrm{model}}}$ via $W^{O}$. This formulation allows the model to jointly attend to information from different representation subspaces at different positions, enriching the learned representation of each token. The design choice to keep the final output dimensionality equal to $d_{\mathrm{model}}$ ensures compatibility with residual connections and layer stacking in transformer architectures.

Cross-Attention. Cross-attention extends the self-attention mechanism to allow one sequence (the *query* source) to attend to another sequence (the *key-value* source). It was introduced in [57] and is particularly important in tasks such as text-to-image generation and text-guided image editing, where the model must condition the output (e.g., an image) on an auxiliary input (e.g., a text prompt). In such settings, the image decoder learns to respond to text embeddings, allowing semantic concepts of the prompt to directly influence the visual output. Modulating the attention maps—by replacing, augmenting, or re-weighting them—enables precise control over spatial layout, geometry, and semantic content of the generated image. Let:

- $Q_{\mathrm{img}}\in R^{n\times d}$ be the matrix of query vectors derived from the image decoder (e.g., latent image tokens).
- $K_{\mathrm{text}},V_{\mathrm{text}}\in R^{m\times d}$ be the key and value matrices derived from the text encoder (e.g., token embeddings), where *m* is the length of the text sequence and *d* is the embedding dimensionality.

The cross-attention operation is defined as:

(37) $$\mathrm{CrossAttention}\left(Q_{\mathrm{img}},K_{\mathrm{text}},V_{\mathrm{text}}\right)=\mathrm{softmax}\left(\frac{Q_{\mathrm{img}}K_{\mathrm{text}}^{⊤}}{\sqrt{d}}\right)V_{\mathrm{text}},$$

where:

- $Q_{\mathrm{img}}K_{\mathrm{text}}^{⊤}\in R^{n\times m}$ contains the pairwise dot-product similarities between image queries and text keys;
- The softmax normalizes each row to a probability distribution over the *m* text tokens;
- The result is a matrix of size $R^{n\times d}$, where each image token is a weighted combination of the text values.

**Figure 13:**
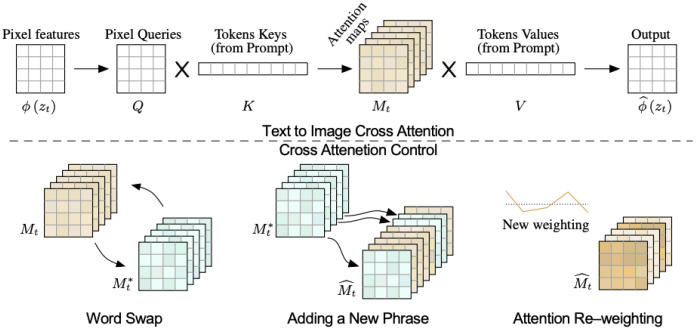
Cross-attention mechanisms. (**Top**) Visual and textual embeddings are combined through cross-attention layers that generate spatial attention maps for each text token. (**Bottom**) The spatial arrangement and geometry of the generated image are guided by the attention maps from a source image. This approach allows various editing tasks to be performed solely by modifying the textual prompt. When replacing a word in the prompt, we insert the source image’s attention maps $M_{t}$, replacing the target image maps $M_{t}^{*}$, to maintain the original spatial layout. Conversely, when adding a new phrase, we only incorporate the attention maps related to the unchanged part of the prompt. Additionally, the semantic influence of a word can be enhanced or reduced by re-weighting its corresponding attention map. Source: [57].

This mechanism enables each spatial or latent position in the image representation to condition its generation on the most relevant tokens from the text prompt. As illustrated in Figure 13, this mechanism enables:

- Semantic alignment: which ensures that visual elements in the output image correspond to the content described in the text;
- Layout preservation: which, by manipulating specific attention maps (e.g., $M_{t}$ for a token *t*), ensures that spatial structure from a reference image can be preserved during editing;
- Prompt-based control: which allows for targeted edits or enhancements when replacing or modifying words in the prompt (which can trigger attention shifts in the image decoder).

Attention mechanisms are a foundational component in language models such as GPT and BERT, where they helps capture contextual relationships within text, and in vision models (e.g., Vision Transformer (ViT) and Diffusion Transformers (DiT in Section 5.4)), where they model spatial relationships between image patches. In generative models such as Stable Diffusion (next Section 5.2.2), self-attention is used within the UNet architecture to enable global spatial dependencies across the image representation. Multi-head attention extends self-attention by enabling the model to project the input into multiple attention heads, each learning to focus on different aspects or subspaces of the data. This mechanism is central to transformer architectures and is used in both text and vision transformers, including generative models such as DALL-E and StyleGAN-T, where diverse and nuanced relationships need to be captured simultaneously across different parts of the input.

Cross-attention, in contrast, involves interactions between different modalities or sequences, where one set of tokens (queries) attends to another set (keys and values). This is crucial in conditional image-generation tasks. For instance, in DALL-E 2 (see Section 5.3) and DALL-E 3, cross-attention allows image representations to attend to text embeddings, enabling coherent image synthesis from textual prompts. Similarly, Stable Diffusion incorporates cross-attention in its denoising network to condition the image-generation process on language inputs.

#### 5.2.2. Latent Diffusion Models (LDMs)

**Figure 14:**
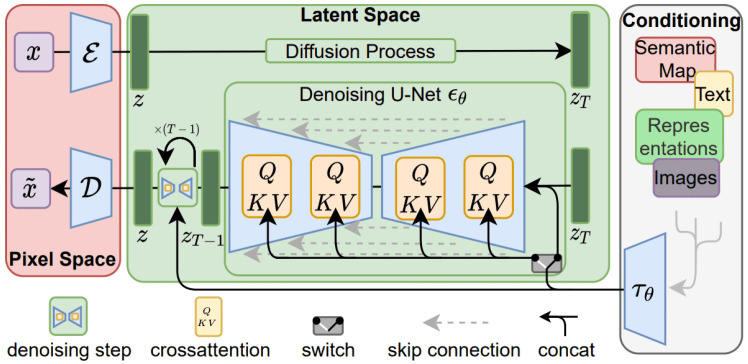
Diagram of the Latent Diffusion Model (LDM) architecture where the input image is encoded into a latent vector z through an encoder E, which will be the input to the forward diffusion process. The denoising U-Net $\epsilon_{\theta }$ utilizes cross-attention layers to process key, query and value pairs (Q, K, V). This setup includes conditioning information through elements such as semantic maps, text and images to guide the transformation back to pixel space using the decoder block D. Source: [45].

Latent Diffusion Models (LDMs) are yet another innovative extension of Diffusion Models [19]. Instead of applying the diffusion on a high-dimensional input (namely pixel or image space), we project the input image into a smaller latent space and apply diffusion with the obtained latents as inputs. The authors of [19] propose to use an encoder network to encode the input into a latent representation and apply the forward process to this latent vector. Then the reverse process is the same as a standard diffusion process with a U-Net to generate new data, which are then reconstructed by a decoder network (see Figure 14). Therefore, given a pre-trained VAE encoder E, which maps an image x to a latent representation z=E(x), the diffusion process is applied in the latent space. The training objective for the Latent Diffusion Model (LDM) is defined as:

(38) $$L_{\mathrm{LDM}}\left(\theta \right)=E_{x,\epsilon ∼N(0,1),\;t}\left[{∥\epsilon -\epsilon_{\theta }\left(z_{t},t\right)∥}_{2}^{2}\right],$$

where the noisy latent $z_{t}$ is generated via the forward diffusion process:

(39) $$z_{t}=\sqrt{{\bar{\alpha }}_{t}}z+\sqrt{1-{\bar{\alpha }}_{t}}\epsilon ,$$

and where:

- z=E(x) is the latent code of input image x;
- ϵ∼N(0,I) is standard Gaussian noise;
- *t* is a timestep sampled uniformly from {1,…,T};
- θ is the learnable parameter;
- $\epsilon_{\theta }\left(z_{t},t\right)$ is the model’s prediction of the noise.

Stable Diffusion can also be conditioned, in particular, using classifier-free guidance by adding conditional embeddings such as image features or text descriptions using a text encoder (e.g., CLIP’s text encoder) to steer the generation process.

### 5.3. Models Combining Diffusion Based Architectures and Transformers

#### 5.3.1. InstructPix2Pix

**Figure 15:**
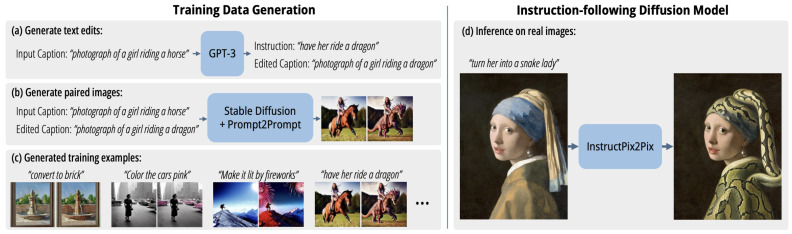
InstructPix2Pix method based on training data generation and Diffusion Model training. (**a**) Fine-tuning GPT-3 to produce editing instructions alongside modified captions. (**b**) These caption pairs are fed into Stable Diffusion with Prompt-to-Prompt guidance to generate corresponding image pairs. (**c**) This process results in a dataset with over 450,000 training samples. (**d**) The authors train the InstructPix2Pix Diffusion Model on this dataset to perform image edits based on textual instructions. During inference, the model can generalize to real-world images and follow human-written editing commands. Source: [58].

Figure 15 illustrates InstructPix2Pix, yet another relevant CGAN-based generative model introduced in 2022 [58] based on the Pix2Pix model [10]. InstructPix2Pix utilizes both LLMs and Diffusion Models by creating a training set consisting of pairs of images and an edit prompt to bridge one image to another. This generated dataset is then used to train a model to generate the resulting images from the input image and edit prompt. Although the dataset is generated, the resulting model is able to generalize and edit input images with arbitrary edit prompts.

#### 5.3.2. DALL-E and DALL-E 2

The first version of DALL-E, introduced by OpenAI in 2021 [59], is a generative model that generates visual outputs given a text description. Training is carried out using a text-image pairs dataset. The architecture of DALL-E is based on a discrete Variational Autoencoder also called a Vector Quantized Variational Autoencoder (VQ-VAE) [60], which maps the input images to image tokens (the VAE mentioned in the section above uses a continuous latent space whereas the (VQ-VAE) uses a discrete latent space). The image and text tokens are concatenated and fed as a single embedding into the network. DALL-E uses an autoregressive transformer (generate one token at a time) to model the joint distribution of text-image pairs (GPT-like Transformer). These generated tokens are converted back into an image via the VQ-VAE decoder. During inference, the target caption is tokenized and concatenated to the output of the (VQ-VAE) and fed to the transformer decoder, which will generate a synthetic image. However, DALL-E showed some limitations due to the discrete tokenization that led to a loss of fine details and lower resolution (256 × 256 images).

A modified version of DALL-E presented as DALL-E 2 in 2022 [61] overcomes these challenges and allows for more complex text inputs, better prompt understanding and more realistic and coherent images. It can also manage high-resolution images, and proposes in-painting (image editing) and out-painting (extending images beyond original borders). The network components of DALL-E 2 varies from DALL-E: instead of a discrete VAE (VQ-VAE), the model uses a Latent Diffusion Model (LDM) as well as a CLIP-based Prior (CLIP: Contrastive Language-Image Pre-training model) that converts text prompts into image embeddings.

CLIP, first introduced by OpenAI in 2021 [62], is a classifier that targets the Natural Language for Visual Reasoning issue by classifying an image into a label (text description of the image) based on its context. It learns to associate images and text descriptions in a shared latent space. In fact, CLIP uses a contrastive learning approach: given text-image pairs, the model learns to maximize the similarity between matching pairs while maximizing the similarity between mismatched pairs.

**Figure 16:**
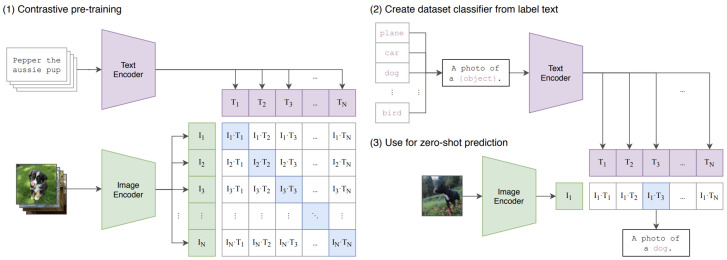
CLIP architecture: CLIP model simultaneously trains an image encoder and a text encoder to correctly match pairs of (image, text) examples within a batch during training. During testing, the trained text encoder produces a zero-shot linear classifier by embedding the names or descriptions of the classes in the target dataset. Source: [62].

**Figure 17:**
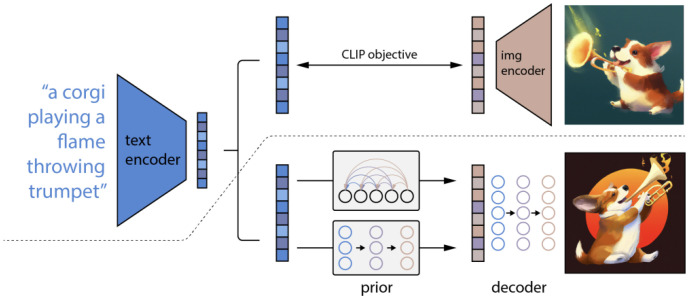
Overview of the DALL-E 2 (or unCLIP) architecture: *Above the dotted line* is illustrated the CLIP training process, which develops a joint representation space for both text and images. *Below the dotted line* is the text-to-image-generation pipeline: a CLIP text embedding is first given as input to an autoregressive or diffusion prior to generate an image embedding, which is then used to condition a diffusion decoder that creates the final image. The CLIP model remains frozen during the training of the prior and the decoder. Source: [61].

This is done by encoding both images and text into vector embeddings using an image encoder network with a text encoder network (see Figure 16, *left side*). The model is trained on large-scale datasets of text-image pairs, enabling it to generalize well to zero-shot learning tasks, meaning it can understand and classify images based on natural language descriptions without task-specific fine-tuning. CLIP’s ability to create meaningful text-image embeddings makes it useful for image-generation application such as DALL-E 2. As illustrated in Figure 17, DALL-E 2 first transforms a text prompt into a CLIP image embedding z using a CLIP prior model p(z|y) where:

- y is the text prompt;
- z is the image embedding in the CLIP latent space;
- p(z|y) is modeled using either a GPT-like Autoregressive Transformer prior or a Diffusion Prior.

Once the CLIP embedding z is obtained, it is passed to a latent Diffusion Model to generate a synthetic image in a lower-dimensional latent space using a pre-trained VAE.

#### 5.3.3. ControlNET

ControlNET was introduced in 2023 [63] and presents an auxiliary network that mirrors U-Net in Stable Diffusion but is conditioned on additional structural guidance (e.g., depth maps, edge maps, or poses). It works by injecting guidance at multiple stages of the U-Net via a set of trainable convolutional layers. These layers receive the conditioning input (e.g., Canny edges) and propagate structured features into the diffusion process, ensuring that the generated image adheres to the input structure while maintaining generative creativity. The guidance information is processed through a zero-convolution module (a 1 × 1 convolution initialized to zero) to ensure smooth integration with the Diffusion Model without destabilizing its pretrained weights.

ControlNET enhances Stable Diffusion by incorporating additional conditioning inputs to guide the image generation process.

#### 5.3.4. Stable unCLIP

Stable unCLIP [64] is a variant of latent Diffusion Models that conditions on CLIP image embeddings in addition to text prompts, enabling effective text-guided image variation and editing tasks. It builds upon the Latent Diffusion Models framework introduced by Rombach et al. [65], extending it to support image-conditioned generation through the use of CLIP embeddings.

Instead of using a text encoder (like OpenAI DALL-E 2’s CLIP or T5) to encode prompts, it takes a CLIP ViT-L/14 image embedding and injects it into the diffusion process as a form of semantic prior. The architecture remains similar to Stable Diffusion, where the U-Net operates in the latent space, guided by the CLIP embedding through cross-attention layers. Additionally, Stable unCLIP employs a learned projection network that maps CLIP image embeddings to Stable Diffusion’s latent space, allowing image variations to be generated without requiring explicit textual guidance. Unlike text-to-image models, which primarily rely on cross-attention with text tokens, Stable unCLIP leverages direct latent conditioning, allowing for greater abstraction in the generated images and for the production of image and text-guided variations at (768 × 768) resolution.

#### 5.3.5. DiffEdit

**Figure 18:**
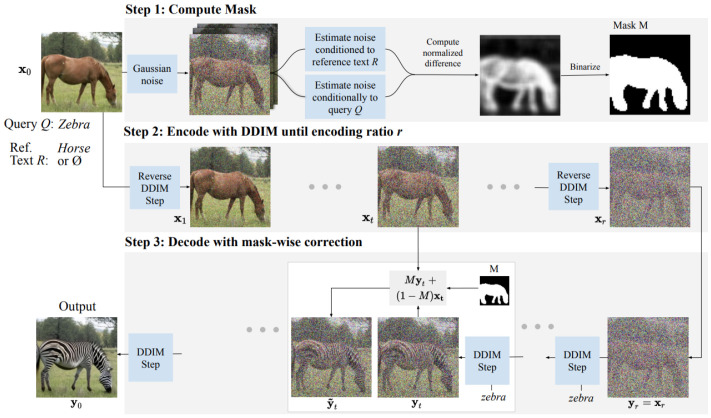
DiffEdit model diagram: first step consists in adding noise to the input image and then denoising it twice—once conditioned on the query text and once conditioned on a reference text (or unconditionally). The differences in the denoising results are used to generate a mask. In the second step, the input image is encoded using DDIM to estimate its latent representation. Finally, in the third step, DDIM decoding is performed conditioned on the text query, with the inferred mask guiding the replacement of the background pixels with values obtained from the encoding process at the corresponding timestep. Source: [66].

First introduced in the paper [66], DiffEdit enhances Stable Diffusion by introducing a mask prediction network that determines which areas of an image should be edited before running the diffusion process. As illustrated in Figure 18, the key innovation here is the dual forward pass through the U-Net:

- First pass: the input image is diffused (noised through forward process) and then denoised using the target text prompt. This provides a preliminary reconstruction of what the model thinks the target image should look like.
- Mask prediction: the difference between the original image and the first-pass reconstruction is computed using a learned discrepancy function, identifying which areas should be modified.
- Second pass (final editing): the identified areas are selectively resampled in the latent space while keeping the unmasked regions frozen, ensuring that only relevant changes are applied.

The core architecture remains that of Stable Diffusion’s latent U-Net, but it incorporates a dynamically computed mask that modifies how noise is applied across different spatial regions. The mask-guided approach prevents unnecessary edits, making it ideal for controlled inpainting and localized modifications.

This approach also ensures that only targeted areas are modified, preserving the rest of the image.

#### 5.3.6. LEDITS++

Introduced in the paper [67], the model LEDITS++ builds upon Stable Diffusion’s latent U-Net while integrating two key additional components: edge-preserving conditioning and CLIP-based semantic guidance. During inference, a source image is first processed to extract its edge representation (typically using a Canny edge detector). These edges are then used as a constraint in the U-Net’s latent space via feature injection layers, which act similarly to ControlNET but with a focus on structural similarity rather than strict adherence to the input. Simultaneously, a CLIP-guided latent optimization step ensures that generated outputs match a target text description while still respecting the original image’s edge structure. The U-Net’s cross-attention mechanism is modified to incorporate both CLIP text embeddings and edge constraints, allowing the Diffusion Model to transform images while preserving spatial features.

### 5.4. Diffusion Transformers (DiT)

One of the most recent diffusion-based models is the Diffusion Transformer (DiT) proposed in [68], which is an architecture that combines the principles of Diffusion Models and transformer models and that generates high-quality synthetic images. It leverages the iterative denoising process inherent in Diffusion Models while utilizing the powerful representation learning capabilities of transformers for improved sample generation. The authors in [68] replace the U-Net backbone, in the LDM model, by a neural network called a Transformer [13]. Transformers are a class of models based on self-attention mechanisms, and they have been proven to excel in tasks involving sequential data (like language processing). They work by attending to all input tokens at once and using multi-head self-attention to process the input efficiently.

**Figure 19:**
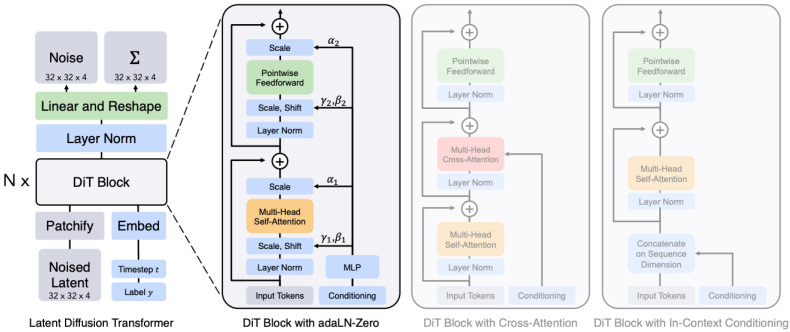
Diffusion Transformer (DiT) architecture: on the left, conditional latent DiT models are trained, where the input latent is divided into patches and processed through multiple DiT blocks. On the right, the DiT blocks include various configurations of standard transformer components that integrate conditioning through methods such as adaptive layer normalization, cross-attention, and additional input tokens. Among these, adaptive layer normalization proves to be the most effective. Source: [68].

In the context of a Diffusion Transformer (see Figure 19), the input to the transformer is typically a set of tokens or features (e.g., image patches, sequence tokens), and self-attention helps the model attend to dependencies across all tokens to capture long-range relationships. In the reverse process of the Diffusion Model, the transformer network is responsible for predicting the noise at each step, conditioned on the noisy data. For example, given the noisy image at time step *t*, the transformer can model long-range spatial dependencies across the image patches (or sequence tokens) and generate a clean image at the next step:

(40) $$x_{t-1}=\mathrm{Transformer}\left(\mu_{\theta }\left(x_{t},t\right),\mathrm{context}\right),$$

where:

- $\mu_{\theta }\left(x_{t},t\right)$ is the predicted noise (as described in the reverse diffusion Equation (31));
- *context* could be a conditioning input, such as a text prompt (in the case of text-to-image generation).

#### DALL-E 3

DALL-E 3 [21] represents the latest advancement in OpenAI’s series of text-to-image generative models, significantly improving the visual fidelity and prompt adherence compared to its predecessor. This model integrates a large-scale language model (GPT-4) with a diffusion-based image-generation pipeline, allowing it to better understand complex textual descriptions and generate images that align closely with the given prompts. DALL-E 3 incorporates an end-to-end approach where the language and image-generation components are deeply coupled, which enhances the model’s ability to faithfully render nuanced details from the text prompt, resulting in higher semantic alignment and image quality.

Although DALL-E 3 uses diffusion-based generation techniques and transformer architectures, it is not explicitly based on the DiT (Diffusion Transformer) architecture. DiTs replace the conventional U-Net backbone in Diffusion Models with pure transformer architectures and achieve strong results in class-conditional image-generation benchmarks such as ImageNet. The key distinction lies in the design objectives: DiT focuses primarily on architectural efficiency and improved Diffusion Model backbones, whereas DALL-E 3 emphasizes the integration of advanced language understanding (via GPT-4) with diffusion to enhance prompt fidelity and user control in image synthesis. Based on that, DALL-E 3 overcomes DALL-E 2’s limitations in handling highly complex prompts by tightly coupling language and image generation through GPT-4 guidance, resulting in more faithful and contextually rich image outputs.

Table 2 summarizes and compares the previously discussed Diffusion and Transformer-based models, highlighting the strengths and limitations of each architecture. While the computational complexity of Diffusion Models can vary depending on architectural choices and implementation details, it is possible to provide a general characterization. The theoretical time complexity can be expressed as:

Training: $O(S\cdot N^{\alpha })$, and Sampling: $O(T\cdot N^{\alpha })$, where:

- *S* is the number of training samples (or batch size);
- *T* is the number of sampling steps required to generate an output;
- *N* is the number of pixels in the input image;
- α is an architecture-dependent exponent, typically α=1 for convolutional networks such as U-Nets, and α=2 for Transformer-based models.

This formulation highlights the key computational scaling properties of Diffusion Models, showing that their cost increases linearly with the number of training examples or sampling iterations, and either linearly or quadratically with image resolution, depending on the model architecture used.

**Table 2:** Comparison of Diffusion Models categorized by methodology, including conditional and latent-guided variants.

Model	Type	Transformer	Pros	Cons
Basic Diffusion Models (DM) [15,16]	Denoising Diffusion Probabilistic Models (DDPM)	No	Simple and stable training; high-quality, diverse outputs.	Slow sampling due to many denoising steps.
Score-Based Generative Models [46]	Score Matching (e.g., SMLD)	No	Theoretically grounded; aligns with likelihood-based training.	High compute; limited control and flexibility.
NCSN [46]	Noise-Conditional Score Networks	No	Trains score functions at multiple noise scales; enables image synthesis from noise.	Requires careful training; lacks intuitive conditioning.
Score-Based Diffusion (SDE) [47]	SDE-based (continuous-time) diffusion	No	Flexible noise schedules; supports fast sampling via ODE solvers.	Complex math; needs denoising score models.
Guided Diffusion [53,69]	Classifier or Classifier-Free Guidance	No	Enables conditional generation with control (class, text, layout, etc.).	Can bias or degrade image quality at high guidance scales.
InstructPix2Pix [58]	Conditional + Guided Diffusion	No	Instruction-guided image editing; strong alignment with user intent.	Requires prompt quality; editing is often limited to style/content described.
ControlNet [63]	Conditional + Guided Diffusion	No	Adds structural control (edges, pose, depth, etc.) to diffusion; high precision.	Heavy model; requires control input (Canny, pose, etc.).
DiffEdit [66]	Masked Conditional Diffusion	No	Local editing with mask guidance; leaves background untouched.	Sensitive to mask boundaries; limited generalization.
LEDITS++ [67]	Localized Conditional Diffusion	No	High-fidelity edits from prompts + structure; state-of-the-art for controllable editing.	Still under research; complex training and model merging.
Stable Diffusion (v1) [65]	Latent Diffusion + Classifier-Free Guidance	No	Efficient and scalable; text-to-image from latent space; open-source.	Harder to train than pixel-space models; prompt sensitivity.
Stable UnCLIP [64]	Latent Diffusion + Image Embedding Conditioned	No	Leverages image embeddings; better reconstruction from reference image.	Reduced diversity; limited to CLIP-space control.
DALL-E 1 [59]	Transformer-based + Discrete Diffusion Decoder	Yes; Autoregressive token generation	Combines VQ-VAE and transformer priors; end-to-end text-to-image.	Coarse outputs; training is complex.
DALL-E 2 [61]	Diffusion Decoder + CLIP Guidance	Yes; Maps text embeddings to image embeddings	High-fidelity images from text; CLIP-based guidance improves alignment.	Prone to prompt leakage or repetition; less open.
DALL-E 3 [21]	Transformer-based Diffusion Model	Yes; Semantic alignment, layout planning and concept binding	Best alignment with complex text; enhanced prompt following.	Closed source; requires Azure/OpenAI backend.

## 6. Experimental Setup

### 6.1. Methods for Comparative Analysis

We define a set of metrics to evaluate the general performance of a model family in image generation. Image quality refers to the level of detail in the generated image. A model with high image quality strictly adheres to the imposed restrictions placed on it while maintaining a high level of detail, and absence of artifacts. A model with low image quality consistently generates images with large amounts of noise and/or artifacts and incoherent features [70]. A model’s diversity refers to its range of potential outputs. A model with high diversity can produce a wide spectrum of images while maintaining a constant image quality. A model with low diversity can only generate images in a narrow range with constant image quality [71]. Leaving this narrow range can lead to significant and rapid decreases in image quality. Controllability refers to the ease with which one can guide the image-generation process with some additional input. For example, if one wanted to generate variations of an image, they could condition the model with an input image to help shape the generated output. A highly controllable model can take into account additional user input, understand the underlying features, and apply those features to the generated image. Training stability refers to the model’s ability to reliably and smoothly converge over the training process.

Within the scope of generative models for image synthesis, Diffusion Models stand out for their ability to produce the highest quality images, often surpassing GANs, which also generate sharp visuals but may not achieve the same level of detail as diffusion-based approaches. VAEs, on the other hand, tend to yield blurrier images, indicating a trade-off in image fidelity. When it comes to diversity, both GANs and Diffusion Models excel at generating a wide variety of outputs, while VAEs can struggle with high variability, limiting their performance in certain applications. In terms of controllability, Diffusion Models offer the most significant level of control over the generation process, allowing for precise adjustments, whereas GANs provide moderate to high control that can vary based on specific architectural choices. However, VAEs exhibit limited tractability, making them less suitable for applications requiring fine-tuned image generation. Lastly, in terms of training stability, VAEs and Diffusion Models are generally more stable during the training process, reducing the likelihood of issues, while GANs often face challenges related to instability and mode collapse, which can hinder their performance and diversity [72]. Table 3 summarizes aspects about image quality, diversity, controllability and training stability.

Scientific images can present details at different scales and in high-resolution as they are often acquired using advanced instruments, e.g., microscopes. In order to generate valuable synthetic images to augment scientific datasets, image quality is expected to be higher than in other domains, such as art. For example, MRI (Magnetic Resonance Imaging) scans of human brains [73] must be both detailed and expressly go through the HIPAA (Health Insurance Portability and Accountability Act) guidelines. The ability to generate brain scans with synthetic MRI represents an invaluable opportunity to create more diverse datasets from a few “approved” images, which could be used by researchers to train models [74,75,76]. The challenge with scientific image generation lies in the Controllability or controlling their generation since pre-existent models are typically trained on data dissimilar to specialized imagery such as microscopy data. If one were to just condition on a single cross-section on a standard GAN or Diffusion Model, then the results would likely be suboptimal. Alternatively, training a model from scratch would require a large dataset, which is actually the motivation for using image generation in the first place. Gathering sufficient amounts of data from experimental settings is often difficult, and sometimes impossible, but without the sufficient quantity to minimize bias and reach convergence during training, the models can be useless. Considering the aforementioned strengths, Diffusion Models are expected to exhibit optimal performance in the synthesis of scientific imagery, as they address each of these criteria.

### 6.2. Selected Models and APIs for Image Generation

To systematically evaluate contemporary generative approaches, we categorize our selected models into three functional domains: (1) image generation from noise or textual input, (2) image translation and semantic variation, and (3) image inpainting with masked guidance. This structure allows us to compare and contrast models not only by task type, but also by underlying architecture—spanning GANs, diffusion-based models, and transformer-based architectures. Our selection aims to provide a representative and balanced overview of the current generative modeling landscape.

**(1) Image Generation from Noise or Textual Prompts.** This category includes models that generate images from random noise or from scratch using language-based prompts. We study DCGAN and StyleGAN, and DALL-E 2 and DALL-E 3 as state-of-the-art transformer-based text-to-image models. DCGAN serves as a classical baseline, illustrating stable GAN training and low-resolution synthesis. In contrast, StyleGAN showcases advanced GAN capabilities, producing high-resolution, photorealistic images with fine-grained latent space control, which is key for disentangled representation learning. On the transformer side, DALL-E 2 and DALL-E 3 represent autoregressive and diffusion-based text-to-image architectures that operate on powerful image-text joint embeddings. We will refer to these models as DALL-E 2 (generation) and DALL-E 3 (generation), respectively. This category collectively enables us to examine unconditional and prompt-based generation, as well as architectural differences in sampling and representation.

**(2) Image Translation and Semantic Variation.** Here, we examine models that take an existing image as input and produce a semantically modified version, often guided by language or structural conditioning. This includes diffusion-based models like Stable unCLIP, LEDITS++, and InstructPix2Pix, as well as the transformer-conditioned diffusion framework ControlNet. Stable unCLIP and LEDITS++ translate an input image based on a target prompt, enabling semantic transformations while preserving content. InstructPix2Pix focuses on instruction-driven edits (e.g., "make circles larger"), demonstrating strong alignment with natural language commands. ControlNet adds an extra layer of structure by introducing a secondary conditioning input such as edge maps or segmentation masks (in our use case, the model uses the Canny edges, i.e., the structural cues, to guide full image generation). Its hybrid design enables spatial control combined with textual semantics. We also evaluate DALL-E 2 (variation mode) which creates semantic variations by taking an input image without prompt guidance and to which we will refer to as DALL-E 2 (variation). This category allows us to probe the boundaries of controllability, latent consistency, and editing capacity in diffusion and transformer-enhanced pipelines.

**(3) Image Inpainting with Masked Edits.** In this final group, the models specialize in localized image editing, where masked regions of an image are filled in, based on surrounding context and semantic prompts. We consider DiffEdit, a diffusion-based mask-aware model, and the DALL-E 2 (edit mode), which supports guided inpainting and to which we will refer to as DALL-E 2 (edit). DiffEdit automatically detects editable regions by contrasting source and target prompts and generating semantic masks, which are refined using a diffusion-based denoising process. DALL-E 2 (edit) allows manual masks and natural language prompts to guide the regeneration of masked areas, ensuring contextual coherence and semantic alignment. These models illustrate the utility of combining image structure and language semantics for fine-grained editing tasks, and they offer insight into localized sampling capabilities within generative frameworks.

Table 4 summarizes models from all three major generative families: GANs (DCGAN, StyleGAN), Diffusion Models (Stable unCLIP, DiffEdit, InstructPix2Pix, ControlNet, LEDITS++), and transformer-based models (DALL-E 2 and 3). By spanning the full range of synthesis tasks, our study provides a comprehensive assessment of how different architectures approach image generation, transformation, and inpainting. This comparative framework enables a deeper understanding of trade-offs in fidelity, controllability, and semantic alignment across model classes.

Regarding computational complexity, Diffusion Models have slower sampling speeds than GANs, but recent advances such as Denoising Diffusion Implicit Models (DDIM) and progressive distillation are helping mitigate runtime while preserving output quality [77].

## 7. Experiments with genAI for Scientific Images

Our experiments consist of using experimental images acquired at LBNL facilities and running a variety of genAI algorithms that aim to mimic key properties present in each of those sets. Datasets range from objects with thin regular structures (fibers) to irregular filaments (roots), to bulk materials (rocks), enabling access to the performance of generative tasks across the spectrum of intensities, textures, sizes, and shapes.

Both the *rocks* and *fibers* datasets are microCT images acquired at the LBNL synchrotron beamline with energies between 10 and 45 keV, with a 1% bandpass, CCD camera Cooke PCO 4000, Kodak chip with 4008 × 2672 pixels, 14 bit, 9 micron square pixels. The image slices come from reconstructions of the parallel beam projection data [78].

**Figure 20:**
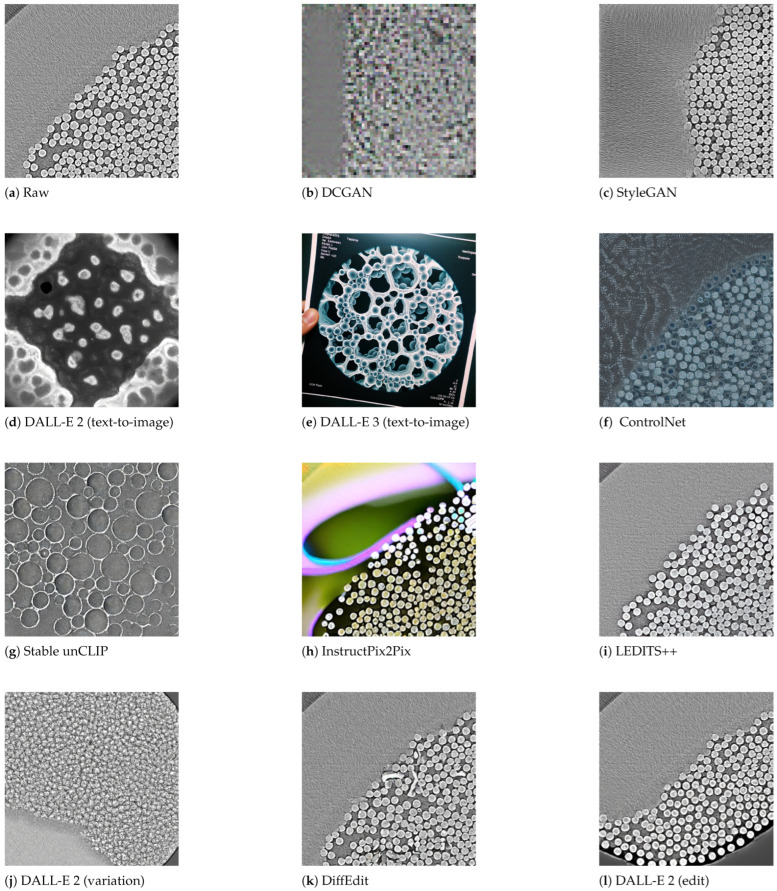
Comparison of image-generation models for the fiber dataset. DCGAN was trained on root images resized to (64,64). DALL-E 2 and DALL-E 3 perform zero-shot image generation from text prompts such as *x-ray image of a composite material with deformed circles* as cross-sections.

**Figure 21:**
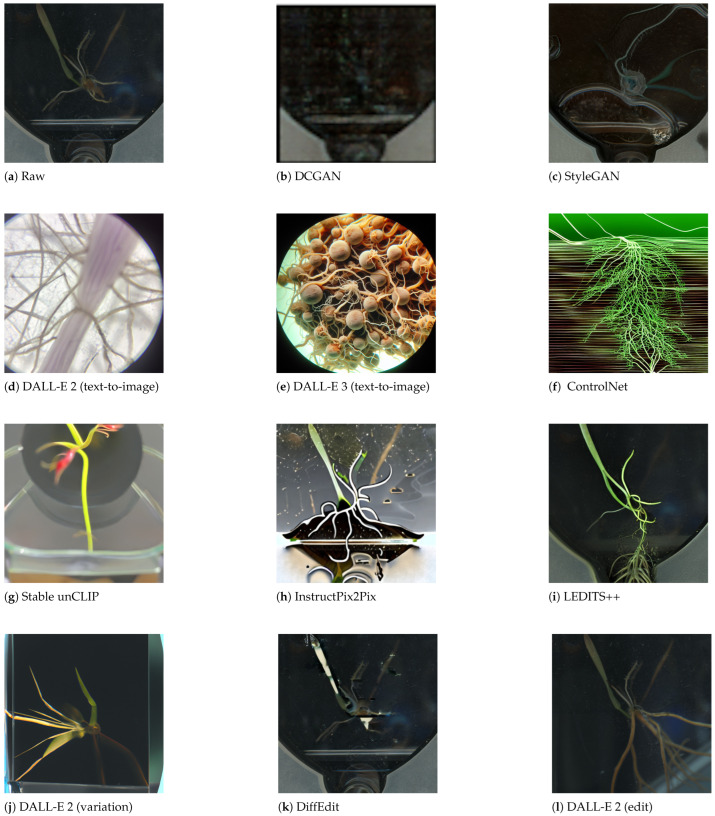
Comparison of image-generation models for the root dataset. DCGAN was trained on root images resized to (64,64). DALL-E 2 and DALL-E 3 perform zero-shot image generation from text prompts such as *microscopy image of entangled plant root in hydroponic system*.

**Figure 22:**
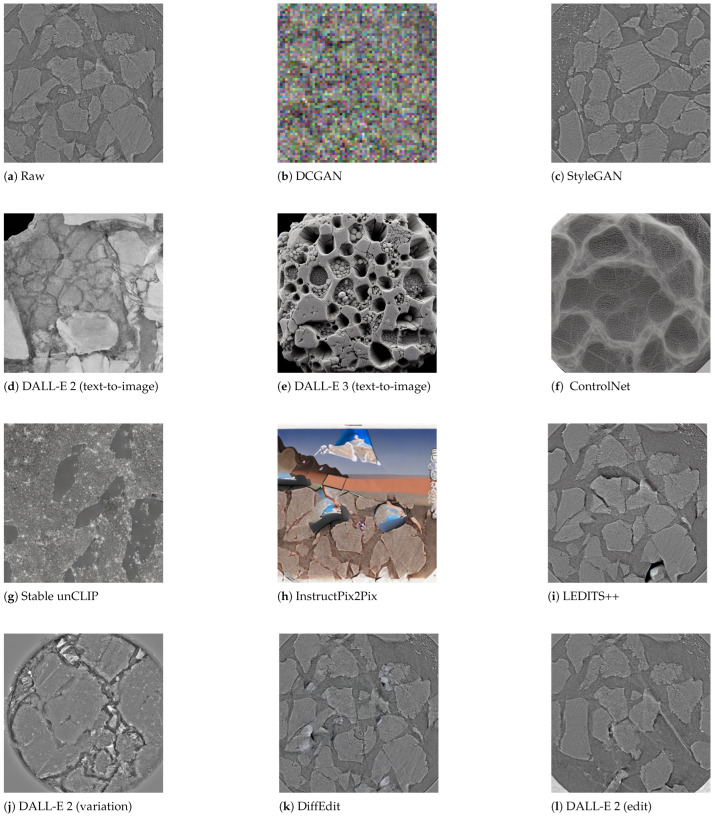
Comparison of image-generation models for the root dataset. DCGAN was trained on rock images resized to (64,64). DALL-E 2 and DALL-E 3 perform zero-shot image generation from text prompts such as *microCT scan of rock sample containing large grains*.

- Fibers: These images come from high-resolution imaging, achieved using synchrotron X-ray radiation to probe the fiber structure and integrity, enabling characterization at the micrometer scale. The reconstructed samples from the parallel-beam projection data are image stacks. These samples are composed of Ceramic Matrix Composites (CMCs), a class of materials engineered to enhance toughness and high-temperature performance compared to monolithic ceramics. This enhancement is achieved by incorporating reinforcement fibers within the ceramic matrix. The interplay between these fibers and the matrix, along with the behavior of the interfaces between them, dictates the overall mechanical properties and the material’s degradation pathways under load [79]. We will refer to this dataset as the *CMC* dataset, which contains 937 high-resolution images of shape (2560,2560). Figure 20 summarizes the experimental results using the selected methods in Table 4.
- Roots: This dataset consists of slices scanned by an automated robotic system called EcoBOT that enables high-throughput scanning of plants in hydroponic systems known as EcoFABs. EcoBOT scans roots using a professional-grade EPSON Perfection V850 Pro scanner for image acquisition. This scanner provides exceptional precision and quality for various media, including paper sheets. Key features include a dual high-resolution lens system (up to 6400 dpi for photos, documents, and 35 mm film/slides) [80]. We will refer to this dataset as the *EcoFAB* dataset, which counts 375 high-resolution images of shape (2039,3000). Figure 21 shows the experimental results.
- Rocks: This dataset comprises microCT scans from samples containing large sediment grains from the Hanford DOE contaminated nuclear site. These sediment grains are contained within a tube, and individual image slices exhibit a visually distinct contrast between the solid grains and the pore space. This dataset has been used for benchmarking segmentation algorithms that separate the pore space from the grains [81]. This is complicated by the presence of reconstruction artifacts, specifically ring artifacts resulting from the back-projection algorithm. Although the inherent contrast between solid and pore space is good, these artifacts introduce streaks that make segmentation difficult. We will refer to this dataset as the *Rocks* dataset, which counts 502 high-resolution images of shape (1813,1830). Figure 22 presents the experimental results.

In these experiments, GAN-based models were trained from scratch for each dataset, whereas diffusion-based models were used purely for inference in their pre-trained state without any task-specific fine-tuning. While high-fidelity outputs were not expected under these conditions, the objective was to assess the baseline performance of state-of-the-art image-generation models when applied to scientific imagery. These inference-only evaluations targeted models that accept either a text prompt (commonly utilized for image editing tasks), an input image, or both. For inference models, between 1 to 3 output images were generated per input, resulting in approximately 1000 to 2000 total images per model. In contrast, for GAN-based models trained from scratch, the number of outputs was directly controlled to generate a similar volume of approximately 2000 images. The dataset comprised a diverse set of scientific images, each accompanied by descriptive metadata. For models constrained to fixed input resolutions, cropped regions of the original images were used to maintain visual fidelity.

In terms of HPC resources, Table 5 provides a comparative summary of computational resources, runtime efficiency, and dataset characteristics for all selected generative models applied to all three image types (CMC, EcoFAB and Rocks). Each table details the model category, GPU configuration, average compiling or inference time, and the number and resolution of images processed. In particular, DCGAN and StyleGAN were trained on each dataset type downsampled or cropped to the appropriate resolutions, whereas API-based models, including DALL-E-based models, were used to perform inference for each high-resolution images. All computations were performed using NVIDIA A100 GPUs. Together, those tables offer insight into the scalability, efficiency, and deployment context of different generative approaches across diverse scientific imaging domains.

### 7.1. Quantitative and Qualitative Results

Verification and validation (V&V) are critical to ensuring the reliability of generative AI models, especially in scientific domains where hallucinations, dataset biases, and lack of ground truth can lead to misleading outputs. Verification focuses on whether the model meets formal specifications, including unit testing and performance benchmarking. Validation assesses whether generated content aligns with real-world phenomena, often relying on both expert judgment and quantitative metrics. SSIM (Structural Similarity Index) evaluates image similarity based on luminance and structural consistency, offering interpretable scores for low-level fidelity. LPIPS (Learned Perceptual Image Patch Similarity) uses deep neural network embeddings to assess perceptual realism, capturing textural and semantic similarity. FID (Fréchet Inception Distance) compares the distribution of real and generated image features, quantifying global realism and diversity. CLIPScore measures semantic alignment between images and text prompts in a joint embedding space, which makes it particularly relevant for prompt-based generation. SSIM and LPIPS operate on image pairs, offering localized evaluations, while FID and CLIPScore evaluate entire image sets. Together, these metrics capture complementary aspects of quality, enabling robust assessment of generative performance. When combined with domain-specific priors and expert validation, they provide a rigorous foundation for the responsible use of GenAI in science. Further information about these metrics are available in Appendix D.

Across the three datasets, we observed that pairwise metrics such as SSIM and LPIPS are not fully representative for unconditional generative models like DCGAN, StyleGAN, and text-to-image models such as DALL-E 2 and DALL-E 3. In fact, these models generate images without direct input-output pairs, making structural and perceptual similarity comparisons, with unrelated reference images, less meaningful. Consequently, for these unconditional models, we primarily rely on FID, which assesses the overall distributional similarity and image realism more robustly. In contrast, for pairwise inference models—ControlNet, LEDITS++, DiffEdit, InstructPix2Pix, Stable unCLIP, DALL-E 2 (edit) and DALL-E 2 (variation)—that produce outputs directly conditioned a specific input image, we evaluate performance using the full set of metrics (SSIM, LPIPS, FID, and CLIPScore) to capture both structural fidelity and perceptual quality. This combined evaluation framework ensures a fair and informative comparison tailored to each model’s generation paradigm.

### 7.2. Results on CMC Dataset

The quantitative evaluation of generative models on the CMC dataset (Figure 20), presented in Table 6, reveals clear differences in performance across architectures. Among all models, DiffEdit, DALL-E 2 (edit), and LEDITS++ deliver the best visual results (results in bold in Table 6), consistent with their strong SSIM, low LPIPS, and low FID scores. DiffEdit notably preserves structural elements, such as the circular fiber boundary, while making targeted edits, reflecting high fidelity and realism. LEDITS++ also maintains structure but introduces a stylized, drawn aesthetic that, while perceptually consistent, sacrifices photorealism.

In contrast, text-to-image DALL-E 2 and 3 (custom prompts), ControlNet, Stable unCLIP, and InstructPix2Pix produce the least realistic outputs. These models often distort key features or generate incoherent and unrealistic edits, which aligns with their poorer LPIPS and FID scores. Interestingly, InstructPix2Pix reports high SSIM but performs poorly visually, indicating it may preserve low-level structure while failing semantically.

CLIPScores are low across all multimodal models—even those generating good images—likely due to CLIP’s poor alignment with scientific image domains like microCT imaging. This underscores the need for domain-adapted embedding models or additional task-specific evaluation metrics. Overall, the combined metrics and visual assessments highlight the strengths of targeted editing models like DiffEdit and the limitations of general-purpose text-to-image systems in scientific contexts.

### 7.3. Results on EcoFAB Dataset

Table 7 presents each evaluation metric and gives insight on each model’s generative performance for the EcoFAB dataset (Figure 21). These quantitative results reveal a complex relationship between metric performance and visual quality for this particular set of images. Although DiffEdit reports the best overall scores—highest SSIM, lowest LPIPS, and lowest FID—its outputs are visually flawed, introducing unnatural black or RGB artifacts on the root structure. This highlights a limitation of conventional metrics, which reward structural similarity even when semantic fidelity is compromised. In contrast, StyleGAN produced the most realistic textures and plausible images, despite lower SSIM and higher LPIPS, though its color palette appeared slightly muted. DALL-E 2 (edit) yielded visually convincing edits, closely aligned with the prompts, though it occasionally exaggerated root branching beyond what is biologically plausible.

Text-to-image models like DALL-E 2 and 3 (custom prompts) underperformed both numerically and visually, often generating images unrelated to the target domain, explaining their poor FID and misaligned CLIPScores. Similarly, models such as ControlNet, Stable unCLIP, and InstructPix2Pix failed to preserve the spatial structure or semantics of the original images, despite moderate scores in some metrics. This mismatch between metrics and actual utility further underscores the limitations of general-purpose evaluation tools like CLIPScore, particularly in scientific domains like EcoFAB, where domain-specific structure and realism are critical.

### 7.4. Results on Rocks Dataset

The quantitative evaluation on the Rocks dataset (Figure 22), presented in Table 8, reveals diverse model performances with clear strengths and weaknesses. DALL-E 2 (edit) achieves the best overall balance, exhibiting high SSIM, low LPIPS, and low FID, indicating strong structural preservation, perceptual similarity, and semantic alignment. Visually, it produces realistic edits that maintain the original image’s content well. DiffEdit scores well on metrics but visually shows minimal structural changes, preserving the input almost identically while introducing some minor unrealistic artifacts, highlighting a disconnection between metric scores and meaningful edits. LEDITS++ offers strong perceptual quality and realism with low FID and LPIPS, though it exhibits slight stylization, suggesting some deviation from strict realism. StyleGAN generates realistic textures, as reflected in its favorable FID.

Conversely, InstructPix2Pix performs poorly visually, introducing many unrealistic colored artifacts, despite moderate quantitative metrics. DALL-E 2 (variation) generates visually realistic images but fails to maintain the original input structure, which reduces its applicability for structure-sensitive tasks. Similarly, Stable unCLIP exhibits weak structural and perceptual consistency with inputs, resulting in poor visual fidelity and moderate metric performance. Models like ControlNet and DALL-E 3 (custom prompt) also struggle to preserve input semantics and produce plausible outputs.

Overall, the results highlight that while editing-focused models like DALL-E 2 (edit) provide the best combination of realism and semantic alignment, many generative models still face challenges preserving fine structural details, especially in domain-specific scientific images.

## 8. Summary and Discussion

The quantitative and qualitative evaluation of GAN-based and diffusion-based generative models across three scientific datasets—CMC, EcoFAB, and Rocks—reveals consistent trends in model performance and challenges specific to scientific image generation. Editing-focused Diffusion Models such as DiffEdit, DALL-E 2 (edit), and LEDITS++, in terms or quantitative results, consistently achieve the best balance of structural fidelity, perceptual quality, and semantic alignment, as reflected in strong SSIM, low LPIPS, and favorable FID scores. However, visual inspection highlights limitations even in top performers: DiffEdit often preserves input structures almost identically but introduces minor unrealistic artifacts, while LEDITS++ shows stylized but less photorealistic outputs.

GAN-based models like StyleGAN generate realistic textures and plausible images, yet their metrics (SSIM, LPIPS) may not fully capture their performance due to the lack of direct input-output pairing, highlighting the limitations of pairwise similarity metrics in unconditional generation scenarios. Notably, models that emphasize realism over diversity, such as StyleGAN or editing models like DALL-E 2 (edit), seem particularly well-suited for applications like slice interpolation or volumetric reconstruction, where generating structurally consistent intermediate slices is essential.

Text-to-image models, including DALL-E 2 and DALL-E 3 with custom prompts, generally underperform both numerically and visually, frequently producing outputs that stray from the target domain or fail to preserve input structure and follow prompt instruction or description. Similarly, models such as ControlNet, Stable unCLIP, and InstructPix2Pix struggle to maintain semantic and spatial consistency, resulting in incoherent or artifact-laden outputs despite sometimes moderate metric scores. In fact, high SSIM or CLIPScores do not always correlate with visual realism or meaningful edits in scientific contexts, indicating a need for domain-adapted evaluation metrics.

The experimental outcomes are primarily shaped by the inherent strengths and limitations of each generative model architecture when applied to scientific image synthesis. Diffusion Models excel in image quality, controllability, and training stability, which allows them to produce highly detailed and structurally faithful images across diverse scientific datasets. Meanwhile, GAN-based models generate sharp and realistic visuals with notable diversity, but often struggle in stability and sometimes lack structural consistency in scientific contexts due to limited controllability and the absence of explicit conditioning mechanisms. Text-to-image models face challenges in scientific applications as their language and vision encoders are typically trained on general content, leading to issues such as semantic drift and poor retention of domain-specific details, which in turn affect output fidelity despite occasionally favorable quantitative metrics. Collectively, these outcomes reflect the broader theoretical understanding that model architecture, controllability, and training objectives directly translate to performance trade-offs in scientific image generation, especially regarding detail preservation, diversity, and alignment with user intent or specialized scientific features.

Despite some success, these generative models sometimes fail to capture domain-specific scientific fidelity due to fundamental limitations in their multi-modal architecture. In text to image and instruction-based models (which in this case includes models such as DALL-E 2, Stable unCLIP, ControlNet and InstructPix2Pix), the CLIP-based text-encoders or similar text encoders are originally trained on web images and lack tuning to scientific language and scientific visual details. In that sense, such misalignment lead to poor semantic guidance, where prompts referencing domain-specific features (e.g., “microCT slice”, “plant roots”, or “composite material”) are either misinterpreted or ignored during generation, resulting in outputs that deviate from scientifically accurate representations. This is called semantic drift, where generated images follow the form of the prompt without maintaining scientific accuracy and fidelity.

Similarly, in image-to-image models, the visual encoders are typically pretrained on natural image datasets (e.g., ImageNet) and not adapted to scientific domains like microCT or high-resolution images of biological components. As a result, they fail to capture or prioritize fine-grained, structurally meaningful details that are essential in scientific imaging tasks—such as cellular boundaries, mineral textures, or biological symmetries. This lack of domain-specific training undermines the model’s ability to generate or edit content with the necessary precision, even when quantitative scores appear adequate.

These challenges can be potentially mitigated through targeted fine-tuning strategies or by leveraging reinforcement learning techniques such as Reinforcement Learning with Human Feedback (RLHF) to better align generative models with domain-specific structural and semantic requirements. Additionally, integrating domain-specific prompt alignment methods, for example, leveraging contrastive learning between scientific image features and tailored textual descriptors, can improve semantic guidance during generation. Methods like Prompt Tuning with Domain-Adaptive Embeddings—where embeddings are adapted or learned specifically on scientific datasets—could enable models to better interpret and generate relevant content from specialized prompts.

Overall, while diffusion-based editing models currently set the benchmark for generating scientifically meaningful images, advancing fine-tuning strategies and developing domain-specific alignment mechanisms will be necessary to overcome existing limitations and enhance multi-modal generative AI performance for scientific image synthesis, in particular in application areas such as materials science imaging.

Building upon the success of generative Diffusion Models in biomedical imaging, ranging from AdaDiff’s ability to overcome domain shifts in MRI reconstruction [82] to unsupervised domain adaptation frameworks for multi-organ segmentation [83], our work takes a complementary approach. Instead of using yet another biomedical dataset, we explore the untapped potential of these methods in energy-centric science. This allows us to investigate their performance and adaptability in a new domain characterized by unique challenges like modality heterogeneity, fine-scale structures, and data scarcity, particularly given the limited to no representation of this data over the web.

## 9. Conclusions and Future Directions

The future of text-to-image and image-to-image technologies promises significant advancements, with profound implications across diverse fields, notably scientific data analysis. We can anticipate continuous refinements in Diffusion Models, leading to hyper-realistic image generation coupled with increasingly granular control over specific attributes and detail. The expectation is that AI models will enable deeper understanding of contextual relationships, and the production of more nuanced and precise visual representations. Additionally, ongoing optimization of algorithms and hardware will yield faster generation times and reduced computational costs, while cloud-based platforms and mobile applications could democratize access to these technologies. A significant trend is the rapid progression of light-weight multimodal models [84,85], with potential improvements in quality and coherence, particularly taking advantage of high-performance computer systems. Finally, AI will increasingly personalize image generation, learning individual user preferences to produce highly tailored visual outputs.

The impact of these technologies on scientific data analysis, particularly with scarce image sets from specialized instruments, will be transformative. AI-driven data augmentation promises to enable the generation of synthetic data to supplement limited datasets, enhancing the training of machine learning models for critical tasks like image segmentation and object detection. In addition, AI will translate abstract scientific data into intuitive visual representations, facilitating the identification of patterns and trends in fields such as genomics and materials science. By generating visual representations of potential scenarios, AI will assist scientists in formulating hypotheses and designing experiments, such as simulating molecular interactions or astronomical phenomena. AI can also be used to identify and rectify errors in scientific images, improving the accuracy and reliability of data analysis. Furthermore, AI will encourage increased collaboration by creating easily understandable visual representations of data for various scientific audiences.

Despite the immense potential, challenges remain. AI models can inherit biases from training data, leading to inaccurate results, which requires careful attention to dataset representativeness. The “black box” nature of some AI models poses challenges to interpretability, requiring efforts to develop more transparent models for scientific applications. Crucially, validation of AI-generated results against experimental data and established scientific principles is essential, especially when dealing with scarce datasets, to ensure the responsible and successful application of these powerful tools.

## Figures and Tables

**Table 1:** Comparison of GAN models by type, architecture, advantages, and limitations.

Model	Type	Pros	Cons
DCGAN [37]	Convolutional GAN	Simple and stable architecture for small datasets; good for learning visual representations; useful baseline for unsupervised generation tasks.	Limited to low-resolution outputs (e.g., 64, 64); prone to mode collapse and training instability; lacks semantic control over outputs.
Pix2Pix [10]	Conditional GAN	Performs high-quality, detailed image-to-image translation when paired data is available; easy to train and fast convergence.	Requires aligned input-output pairs; not applicable to unpaired settings; limited output diversity.
CycleGAN [11]	Conditional GAN	Enables unpaired image translation using cycle consistency; works well with domain adaptation and style transfer without paired data.	Poor at handling large domain gaps; produces deterministic outputs; sensitive to cycle loss weighting.
StackGAN [41]	Conditional GAN	Generates high-res images from text using two-stage coarse-to-fine refinement; improves detail and realism.	Complex training pipeline; intermediate outputs may be poor; struggles with long or complex text prompts.
AttnGAN [42]	Conditional GAN	Uses attention mechanisms for word-region alignment; improves text-to-image coherence and fine detail generation.	Heavy computation and sensitive to noise in attention; can overfit; complex to tune and interpret.
StyleGAN [12]	Style-Based GAN	Generates photorealistic images with disentangled control over features like age or pose; smooth latent space for editing.	Requires large, clean datasets and high compute; expensive training; earlier versions had visual artifacts.
GigaGAN [39]	Style-Based GAN	Combines GAN speed with diffusion-level quality; supports text-to-image at high resolution (e.g., 1024,1024); fast inference.	Requires massive compute and data; complex architecture; difficult to stabilize and reproduce training.

**Table 3:** Comparison of VAEs, GANs, and Diffusion Models for text-to-image generation.

Model Type	Image Quality	Diversity	Controllability	Training Stability
Variational Autoencoders (VAEs)	Moderate to High: Generally produces images with good quality but can be blurry due to the loss function used.	Moderate: Capable of generating diverse images but may struggle with high variability in complex datasets.	Moderate: Can condition on text embeddings but lacks fine-grained control over image features.	High: More stable during training compared to GANs, but can suffer from issues such as posterior collapse.
Generative Adversarial Networks (GANs)	High: Known for generating sharp and detailed images.	High: Capable of producing a wide variety of images, especially with diverse training data.	Moderate to High: Can implement various conditioning methods (e.g., text-to-image) but may require complex architectures for precise control.	Moderate: Training can be unstable and sensitive to hyperparameters; mode collapse can occur, leading to reduced diversity.
Diffusion Models	Very High: Achieves state-of-the-art image quality, often surpassing GANs and VAEs in realism and detail.	High: Generates diverse images effectively, with the potential for high variability.	High: Allows for more explicit control over the generation process through iterative denoising steps and conditioning.	Moderate: High time and space complexity but generally more stable than GANs during training, with well-defined training objectives that reduce issues such as mode collapse.

**Table 4:** Summary of selected generative models grouped by functional domain, architecture, and key features.

Domain	Model	Architecture	Description / Key Features
Image Generationfrom Noise or Text	DCGAN	GAN	Classical baseline; stable training; low-res synthesis.
	StyleGAN	GAN	High-res, photorealistic images with fine-grained latent space control.
	DALL-E 2 (gen)	Transformer + Diffusion	Autoregressive text-to-image using joint image-text embeddings.
	DALL-E 3 (gen)	Transformer + Diffusion	Improved alignment and semantic comprehension over DALL-E 2.
Image Translationand Semantic Variation	Stable unCLIP	Diffusion	Prompt-based translation; preserves image structure.
	LEDITS++	Diffusion	Guided semantic editing with strong content retention.
	InstructPix2Pix	Diffusion	Text instruction-based edits; fine-grained control.
	ControlNet	Diffusion + Transformer	Adds structure (e.g., edges) for spatial control in generation.
	DALL-E 2 (var)	Transformer + Diffusion	Prompt-free semantic variations from input image.
Image Inpaintingwith Masked Edits	DiffEdit	Diffusion	Mask-aware editing via prompt contrast and semantic masks.
	DALL-E 2 (edit)	Transformer + Diffusion	Manual masking with prompt-driven inpainting.

**Table 5:** Overview of computational resources, compilation times, and dataset sizes used for training or inference across all generative models.

Model Category	HPC Resource	Average Compilation Time Per Iteration	Dataset size Trained or Inferred On
DCGAN-Training	1 NVIDIA A100 GPU	40 min	Train on 502 (64,64) images
StyleGAN-Training	4 NVIDIA A100 GPUs	4 h 30 min	Train on 502 (512,512) images
Diffusion Model APIs-Inference	1 NVIDIA A100 GPU	4 to 8 min per iteration	Infer a (512,512) image
DALL-E APIs-Inference	1 NVIDIA A100 GPU	between 10 to 15 s	Infer a (512,512) image

**Table 6:** Quantitative evaluation of generative models on the CMC dataset across multiple tasks, including: (i) image generation from noise or text input, (ii) image-to-image translation and semantic variation, and (iii) masked image editing. Reported metrics include SSIM, LPIPS, and CLIPScore (when applicable), presented as mean ± standard deviation. FID is reported as a single score computed over the distribution of real and generated images. For text-conditoned models, CLIPScore is included to assess semantic alignment with the input prompt. All input and output images correspond to the examples shown in Figure 20 and use consistent image sizes per task, except for DCGAN (trained on resized inputs of (64,64)), and DALL-E 3 (which internally upsamples inputs from (512,512) to (1024,1024) during generation).

Model	SSIM	LPIPS	FID	CLIPScore
DCGAN	0.067 ± 0.008	0.466 ± 0.032	269.848	N/A
**StyleGAN**	**0.039 ± 0.014**	**0.417 ± 0.083**	**77.374**	N/A
DALL-E 2, Custom Prompt	0.043 ± 0.024	0.723 ± 0.085	393.291	0.312 ± 0.027
DALL-E 3, Custom Prompt	0.027 ± 0.013	0.694 ± 0.088	331.425	0.322 ± 0.025
ControlNET	0.037 ± 0.020	0.594 ± 0.103	257.830	0.287 ± 0.033
InstructPix2Pix	0.343 ± 0.091	0.409 ± 0.126	189.967	0.212 ± 0.026
Stable unCLIP	0.031 ± 0.018	0.655 ± 0.111	265.238	0.269 ± 0.025
**LEDITS++**	**0.567 ± 0.078**	**0.092 ± 0.070**	**46.332**	**0.279 ± 0.016**
DALL-E 2 (variation), No Prompt	0.049 ± 0.009	0.373 ± 0.103	240.152	N/A
**DiffEdit**	**0.664 ± 0.076**	**0.053 ± 0.044**	**39.945**	**0.227 ± 0.022**
**DALL-E 2 (edit), Custom Prompt**	**0.523 ± 0.006**	**0.118 ± 0.035**	**41.305**	**0.328 ± 0.016**

**Table 7:** Quantitative evaluation of generative models across different tasks for the EcoFAB dataset, including: (i) image generation from noise or text input, (ii) image-to-image translation and semantic variation, and (iii) masked image editing. Reported metrics include SSIM, LPIPS, and CLIPScore (when applicable), presented as mean ± standard deviation. FID is reported as a single score computed over the distribution of real and generated images. For text-conditoned models, CLIPScore is included to assess semantic alignment with the input prompt. All input and output images correspond to the examples shown in Figure 21 and models follow similar specifications as described in Table 6.

Model	SSIM	LPIPS	FID	CLIPScore
DCGAN	0.067 ± 0.008	0.466 ± 0.032	305.426	N/A
**StyleGAN**	**0.233 ± 0.020**	**0.607 ± 0.028**	**90.520**	N/A
DALL-E 2, Custom Prompt	0.282 ± 0.048	0.695 ± 0.036	405.784	0.345 ± 0.021
DALL-E 3, Custom Prompt	0.131 ± 0.027	0.664 ± 0.034	302.775	0.301 ± 0.031
ControlNET	0.085 ± 0.037	0.820 ± 0.041	380.363	0.276 ± 0.056
Stable unCLIP	0.391 ± 0.034	0.654 ± 0.035	218.497	0.251 ± 0.040
InstructPix2Pix	0.520 ± 0.062	0.428 ± 0.075	143.595	0.261 ± 0.043
LEDITS++	0.673 ± 0.056	0.343 ± 0.041	206.072	0.316 ± 0.028
DALL-E 2 (variation), No Prompt	0.566 ± 0.069	0.578 ± 0.035	178.433	N/A
DiffEdit	0.843 ± 0.046	0.147 ± 0.030	64.686	0.183 ± 0.036
**DALL-E 2 (edit), Custom Prompt**	**0.751 ± 0.028**	**0.293 ± 0.019**	**189.235**	**0.306 ± 0.031**

**Table 8:** Quantitative evaluation of generative models across different tasks for the Rocks dataset, including: (i) image generation from noise or text input, (ii) image-to-image translation and semantic variation, and (iii) masked image editing. Reported metrics include SSIM, LPIPS, and CLIPScore (when applicable), presented as mean ± standard deviation. FID is reported as a single score computed over the distribution of real and generated images. For text-conditoned models, CLIPScore is included to assess semantic alignment with the input prompt. All input and output images correspond to the examples shown in Figure 22 and models follow similar specifications as described in Table 6.

Model	SSIM	LPIPS	FID	CLIPScore
DCGAN	0.067 ± 0.008	0.466 ± 0.032	269.848	N/A
**StyleGAN**	**0.233 ± 0.017**	**0.693 ± 0.018**	**69.51**	N/A
DALL-E 2, Custom Prompt	0.155 ± 0.032	0.532 ± 0.028	292.503	0.311 ± 0.018
DALL-E 3, Custom Prompt	0.069 ± 0.019	0.586 ± 0.027	329.988	0.293 ± 0.019
ControlNET	0.240 ± 0.044	0.636 ± 0.061	368.932	0.295 ± 0.026
StableunCLIP	0.216 ± 0.023	0.515 ± 0.027	313.798	0.297 ± 0.022
InstructPix2Pix	0.443 ± 0.046	0.370 ± 0.081	169.820	0.274 ± 0.031
**LEDITS++**	**0.440 ± 0.015**	**0.161 ± 0.019**	**65.451**	**0.294 ± 0.011**
DALL-E 2 (variation), No Prompt	0.191 ± 0.046	0.488 ± 0.035	283.140	N/A
DiffEdit	0.556 ± 0.011	0.135 ± 0.029	124.199	0.276 ± 0.011
**DALL-E 2 (edit), Custom Prompt**	**0.635 ± 0.012**	**0.216 ± 0.015**	**75.104**	**0.314 ± 0.009**

## Data Availability

Data and code supporting reported results can be found at https://github.com/lbl-camera/genAI upon acceptance and will include links to publicly archived datasets analyzed or generated during the study for reproducibility (accessible on 15 July 2025).

## Appendix A. Mathematical Details on CycleGAN Loss

We follow the formulation introduced in the paper [11], which uses adversarial losses and a cycle consistency loss to enable unpaired image-to-image translation.

For generator *G* and discriminator $D_{Y}$, the **adversarial loss** is:

(A1) $$L_{\mathrm{GAN}}\left(G,D_{Y},X,Y\right)=E_{y∼p_{\mathrm{data}}\left(y\right)}\left[\log D_{Y}\left(y\right)\right]+E_{x∼p_{\mathrm{data}}\left(x\right)}\left[\log\left(1-D_{Y}\left(G\left(x\right)\right)\right)\right].$$

Similarly, for generator *F* and discriminator $D_{X}$:

(A2) $$L_{\mathrm{GAN}}\left(F,D_{X},Y,X\right)=E_{x∼p_{\mathrm{data}}\left(x\right)}\left[\log D_{X}\left(x\right)\right]+E_{y∼p_{\mathrm{data}}\left(y\right)}\left[\log\left(1-D_{X}\left(F\left(y\right)\right)\right)\right].$$

To ensure that the learned mappings are meaningful and reversible, CycleGAN introduces a **cycle consistency loss**:

(A3) $$L_{\mathrm{cyc}}\left(G,F\right)=E_{x∼p_{\mathrm{data}}\left(x\right)}\left[{∥F\left(G\left(x\right)\right)-x∥}_{1}\right]+E_{y∼p_{\mathrm{data}}\left(y\right)}\left[{∥G\left(F\left(y\right)\right)-y∥}_{1}\right].$$

## Appendix B. Background on Denoising Diffusion Probabilistic Models (DDPMs)

The *forward* process in the diffusion network consists of a Markov chain of *T* steps. Given an input image $x_{0}∼q\left(x_{0}\right)$, Gaussian noise is added at each step t<T according to a variance schedule $\beta_{1},\ldots ,\beta_{T}$:

(A4) $$\begin{array}{cc} q(x_{t}|x_{t-1}) & =N(x_{t};\sqrt{1-\beta_{t}}x_{t-1},\beta_{t}I) \end{array}$$

(A5) $$\begin{array}{cc} \; & =\sqrt{1-\beta_{t}}x_{t-1}+\sqrt{\beta_{t}}\epsilon , \end{array}$$

with ϵ∼N(0,I).

Using the reparametrization trick and defining $\alpha_{t}=1-\beta_{t}$, ${\bar{\alpha }}_{t}=\prod_{s=1}^{t}\alpha_{s}$, the authors [16] derive:

(A6) $$\begin{array}{cc} q(x_{t}|x_{0}) & =N(x_{t};\sqrt{{\bar{\alpha }}_{t}}x_{0},\left(1-{\bar{\alpha }}_{t}\right)I) \end{array}$$

(A7) $$\begin{array}{cc} \; & =\sqrt{{\bar{\alpha }}_{t}}x_{0}+\sqrt{1-{\bar{\alpha }}_{t}}\epsilon . \end{array}$$

The joint posterior of the forward process is:

(A8) $$q\left(x_{1:T}|x_{0}\right)=\prod_{t=1}^{T}q\left(x_{t}|x_{t-1}\right).$$

The reverse process is also modeled as a Markov chain:

(A9) $$\begin{array}{cc} p_{\theta }\left(x_{t-1}|x_{t}\right) & =N(x_{t-1};\mu_{\theta }\left(x_{t},t\right),\Sigma_{\theta }\left(x_{t},t\right)) \end{array}$$

(A10) $$\begin{array}{cc} p_{\theta }\left(x_{0:T}\right) & =p_{\theta }\left(x_{T}\right)\prod_{t=1}^{T}p_{\theta }\left(x_{t-1}|x_{t}\right), \end{array}$$

with $p\left(x_{T}\right)=N\left(0,I\right)$.

## Appendix C. Mathematical Details of Score-Based SDEs

Let the formulations for the Variance Preserving and the Variance Exploding be:

- In the Variance Preserving (VP) formulation, the coefficients are set as $f\left(t\right)=-\frac{1}{2}\beta \left(t\right)$ and $g\left(t\right)=\sqrt{\beta (t)}$, where β(t) is a time-dependent variance schedule.
- Alternatively, the Variance Exploding (VE) formulation employs f(t)=0 and a monotonically increasing diffusion term g(t)=σ(t), often chosen to grow exponentially.

These choices of SDE formulations and corresponding noise schedules constitute a key part of the model-design space and have been further studied for sample quality, likelihood estimation, and computational efficiency [49]. The conditional distribution of the noisy variable x(t) given the clean input x(0) is Gaussian (defined in Equations (11) and (12) of [49]).

(A11) $$p_{0t}\left(x\left(t\right)|x\left(0\right)\right)=N\left(x\left(t\right);\;s\left(t\right)x\left(0\right),\;\sigma^{2}\left(t\right)I\right),$$

where the scale and variance functions are defined using *f* and *g* by the following:

(A12) $$s\left(t\right)=\exp\left(\int_{0}^{t}f\left(\xi \right)\;d\xi \right),\;\sigma^{2}\left(t\right)=s^{2}\left(t\right)\int_{0}^{t}\frac{g^{2}\left(\xi \right)}{s^{2}\left(\xi \right)}\;d\xi .$$

## Appendix D. Verification and Validation

Hallucinations and unexpected outcomes are some of the issues associated with GenAI. Other problems include inherent biases within training datasets that can skew the generated images, reinforcing existing misconceptions [5] or overlooking important, yet underrepresented, scientific phenomena. Validation becomes exceptionally difficult when dealing with completely novel scenarios, as there may be no existing experimental or observational data for comparison. This lack of ground truth poses the risk of generating misleading visualizations that could inadvertently guide research down unproductive paths; therefore only rigorous scrutiny and expert validation could potentially mitigate these risks [6].

Verification and Validation (V&V) are essential for establishing the reliability and accuracy of AI generative models, and several efforts have focused on creating standardized benchmarks [84]; however, curated datasets using scientific imaging are either extremely narrow [86,87,88] or sparse [89,90]. Verification assesses a model’s adherence to specified requirements and its performance under defined conditions. This includes unit testing for component correctness and performance evaluation against benchmark datasets. Cross-validation further examines the predictive performance across data subsets, indicating robustness. Validation determines whether the model accurately reflects real-world phenomena. In scientific imaging, validation involves qualitative expert (domain scientist) evaluations of generated image realism and quantitative metrics.

To evaluate the quality of generated images, a combination of complementary quantitative metrics is used. **SSIM (Structural Similarity Index Measure)** captures structural and textural similarities between the generated and reference images, aligning well with human visual perception. **LPIPS (Learned Perceptual Image Patch Similarity)** further evaluates perceptual similarity by comparing deep features extracted from neural networks, providing a learned measure of visual realism. Beyond pairwise comparisons, **FID (Fréchet Inception Distance)** quantifies the distance between distributions of real and generated image features, offering a global assessment of fidelity and diversity. Lastly, **CLIPScore** measures semantic alignment between generated images and textual prompts using multimodal embeddings, making it particularly relevant for prompt-conditioned generative tasks. Together, these metrics offer a comprehensive evaluation across perceptual similarity, statistical fidelity, diversity, and semantic alignment.

SSIM and LPIPS are pairwise image similarity metrics, computed between corresponding real and generated images (that will be denoted as *x* and $\hat{x}$ respectively and with heights H and width W), providing localized assessments of structural and perceptual fidelity. In contrast, FID and CLIPScore are set-level metrics that operate over sets of images to quantify distributional divergence (FID), realism and diversity (FID) and alignment between image content and textual prompts (CLIPScore). We place particular emphasis on these metrics as they are widely accepted for evaluating generative models, providing a mix of objective accuracy and perceptual quality measures.

- **Structural Similarity Index (SSIM)** SSIM compares local patterns of pixel intensities (luminance, contrast, structure):
  (A13) $$\begin{array}{c} \mathrm{SSIM}\left(x,\hat{x}\right)=\frac{\left(2\mu_{x}\mu_{\hat{x}}+C_{1}\right)\left(2\sigma_{x\hat{x}}+C_{2}\right)}{\left(\mu_{x}^{2}+\mu_{\hat{x}}^{2}+C_{1}\right)\left(\sigma_{x}^{2}+\sigma_{\hat{x}}^{2}+C_{2}\right)}, \end{array}$$
  where μ, $\sigma^{2}$, and $\sigma_{x\hat{x}}$ denote respectively the mean, variance and covariance of the images, and $C_{1}$, $C_{2}$ are constants for numerical stability. Values range between −1 and 1 in theory and between 0 and 1 in practice, with values closer to 0 showing high level of dissimilarity and values closer to 1 showing excellent reconstruction and perfect similarity in terms of perceived structure.
- **Learned Perceptual Image Patch Similarity (LPIPS)** LPIPS uses a neural network to assess perceptual similarity:
  (A14) $$\begin{array}{c} \mathrm{LPIPS}\left(x,\hat{x}\right)=\sum_{l}\frac{1}{H_{l}W_{l}}\sum_{h,w}{∥{\hat{y}}_{hw}^{l}-y_{hw}^{l}∥}_{2}^{2} \end{array}$$
  where $y^{l}$ and ${\hat{y}}^{l}$ are the normalized feature maps at layer *l* of a pre-trained network and $H_{l}$ and $W_{l}$ are their spatial dimensions. These deep features captures differences in texture, semantics and spatial structure and values range from 0 to 1 and a lower value highlighting very close perceptual features and values closer to 1 showing important perceptual differences.
- **Fréchet Inception Distance (FID)** FID quantifies the distance between the distributions of real and generated image features extracted by the Inception network. It assumes both feature distributions are multivariate Gaussians.
  (A15) $$\begin{array}{c} \mathrm{FID}={∥\mu_{r}-\mu_{g}∥}_{2}^{2}+\mathrm{Tr}\left(\Sigma_{r}+\Sigma_{g}-2{\left(\Sigma_{r}\Sigma_{g}\right)}^{1/2}\right), \end{array}$$
  where $\left(\mu_{r},\Sigma_{r}\right)$ and $\left(\mu_{g},\Sigma_{g}\right)$ denote the mean and covariance of real and generated image features, respectively. Lower FID indicates that the generated images are closer in distribution to real images.
- **CLIPScore** CLIPScore measures the semantic alignment between an image and a text prompt using the CLIP model’s joint image-text embedding space.
  (A16) $$\begin{array}{c} \mathrm{CLIPScore}\left(x,t\right)=\cos\left(\theta \right)=\frac{f_{I}{\left(x\right)}^{⊤}f_{T}\left(t\right)}{∥f_{I}\left(x\right)∥\cdot ∥f_{T}\left(t\right)∥}, \end{array}$$
  where $f_{I}\left(x\right)$ is the normalized image embedding and $f_{T}\left(t\right)$ be the normalized text embedding for image *x* and text *t*. The score reflects cosine similarity between image and text embeddings and thus higher values indicate better semantic alignment.

Incorporating domain-specific knowledge strengthens reliability. For example, in biological or material sciences imaging, comparisons against existing scientific models and datasets ensure that generated outputs are both visually and scientifically sound. Through rigorous V&V, researchers can avoid major pitfalls of generative AI models and potentially model utilization in critical scientific applications.
